# Supplementary material for: A coarse-grained bacterial cell model for resource-aware analysis and design of synthetic gene circuits
Source: Nat Commun. 2024 Mar 4;15:1981. doi: 10.1038/s41467-024-46410-9 (PMC10912777; doi:10.1038/s41467-024-46410-9)
Supplement: Supplementary file 1 — Supplementary Information [file 41467_2024_46410_MOESM1_ESM.pdf]

Supplementary Information: A coarse-grained bacterial cell model for resource-aware analysis and design of synthetic gene circuits

Kirill Sechkar[1] Harrison Steel[1] Giansimone Perrino[2,3,*]
Guy-Bart Stan[2,3,**]

[1]Department of Engineering Science, University of Oxford,
Parks Road, Oxford OX1 3PJ, UK
[2]Department of Bioengineering, Imperial College London,
South Kensington Campus, London SW7 2AZ, UK
[3]Imperial College Centre of Excellence in Synthetic Biology, Imperial College London,
South Kensington Campus, London SW7 2AZ, UK

[*]Corresponding author. Email: g.perrino@imperial.ac.uk
[**]Corresponding author. Email: g.stan@imperial.ac.uk

Supplementary Notes

S1 Model definition **4**

S1.1 Unsimplified mechanistic cell model with flux- parity regulation 4

S1.2 The quasi-steady-state approximation 9

S1.3 Neglecting the mRNA-Ribosome complex dilution 9

S1.4 Cell model with the QSS approximation 11

S1.5 Neglecting the housekeeping genes 12

S1.6 Nutrient upshift modeling 14

S2 Parameter fitting **19**

S2.1 Modeling ribosome inactivation 19

S2.2 MCMC fitting 21

S2.3 Scaling the promoter strengths 24

S3 Heterologous gene expression modeling **29**

S3.1 Extending the mechanistic cell model 29

S3.2 Modeling degradation of heterologous proteins 31

S3.3 Stochastic modeling of heterologous gene expression 32

S3.4 Effect of heterologous gene expression on translation elongation and ribosomal gene transcription rates 34

S3.5 Estimating steady-state protein mass fractions with heterologous expression 38

S3.6 Estimating steady-state growth rate with heterologous gene expression . . 40

S3.7 Maximizing heterologous protein production 41

S4 Biocircuit modeling **44**

S4.1 Feasible synthetic gene parameter ranges 44

S4.2 Two bistable switches exhibiting "winner-takes-all" behavior 45

S4.2.1 Description 45

S4.2.2 Additional simulations 46

S4.3 A bistable non-cooperative self-activator 50

S4.3.1 Description 50

S4.3.2 Additional simulations 51

S4.4 An integral feedback controller for mitigating burden 55

S4.4.1 Description 55
S4.4.2 Estimating the controller's setpoints and operation range 59
S4.4.3 Additional simulations 62

S1 Model definition

In this section, we derive the simplified model of the host bacterial cell, depicted in Figure 2 and defined by Equations (15)-(20) in the main text's Methods section. To this end, we first define a coarse-grained mechanistic cell model that explicitly considers the competitive binding of ribosomes by different mRNAs present in the cell, then reduce the number of considered variables by using the Quasi-Steady-State (QSS) approximation. In order to further simplify the model, we then show how it is possible to omit the Ordinary Differential Equations (ODEs) describing the expression of one of the gene classes into which we partition the bacterium's genome. Finally, we demonstrate how our framework can be extended to capture the cell's reaction to changes in the culture medium's, comparing our model's predictions with experimental data.

S1.1 Unsimplified mechanistic cell model with flux- parity regulation

Our model does not take into account transcriptional resource couplings, as they are believed to have a negligible effect on gene expression [1]. Hence, we focus on the competitive binding of a finite pool of ribosomes by mRNAs. We consider three types of native genes in the bacterial cell: metabolic (a), ribosomal (r), and housekeeping (q). For the gene $i \in \{q, a, r\}$, m_i signifies the concentration of its free transcript, b_i the concentration of its mRNA-ribosome complex, and p_i the concentration of the corresponding protein (the number of amino acid residues in one molecule of protein p_i is denoted as n_i). However, since ribosomes can be either free or bound by an mRNA, instead of modeling the overall concentration of ribosomal protein p_r, we adopt a more specific approach for them, denoting the concentration of non-translating ribosomes by r. Therefore, the total ribosome concentration can be calculated from the modeled variables as

$$R = r + \sum_{j \in \{q,a,r\}} b_j \tag{S1}$$

Additionally, we look at the pool of protein precursors – aminoacylated (charged) tRNA molecules t^c – as well as uncharged tRNAs t^u. The conversion of t^u into t^c is catalyzed by the metabolic proteins p_a. The set of all reactions that we model is therefore

as follows:

- **Transcription:** $\varnothing \xrightarrow{F_i \alpha_i c_i \lambda} m_i$ for $i \in \{q, a, r\}$
- **mRNA degradation and dilution:** $m_i \xrightarrow{\beta_i + \lambda} \varnothing$ for $i \in \{q, a, r\}$
- **mRNA-ribosome binding:** $m_i + r \underset{k_i^+}{\overset{k_i^-}{\rightleftharpoons}} b_i$ for $i \in \{q, a, r\}$
- **mRNA-ribosome complex dilution:** $b_i \xrightarrow{\lambda} \varnothing$ for $i \in \{q, a, r\}$
- **Translation:** $b_i + n_i \times t^c \xrightarrow{\epsilon/n_i} m_i + r + p_i + n_i \times t^u$ for $i \in \{q, a\}$
  but denoted as $b_r + n_r \times t^c \xrightarrow{\epsilon/n_r} m_r + 2 \times r + n_r \times t^u$ for ribosomal genes
- **Protein dilution:** $p_i \xrightarrow{\lambda} \varnothing$ for $i \in \{q, a\}$
  but denoted as $r \xrightarrow{\lambda} \varnothing$ for ribosomes
- **tRNA synthesis:** $\varnothing \xrightarrow{\psi} t^u$
- **tRNA charging (aminoacylation):** $t^u \xrightarrow{\nu p_a} t^c$
- **tRNA dilution:** $t^u \xrightarrow{\lambda} \varnothing$ and $t^c \xrightarrow{\lambda} \varnothing$

These reactions give rise to the following ODE model for the dynamics of the species' concentrations:

$$\dot{m_i} = F_i c_i \alpha_i \lambda(\epsilon, B) - (\beta_i + \lambda(\epsilon, B)) m_i - k_i^+ m_i r + k_i^- b_i + \frac{\epsilon(t^c)}{n_i} b_i \quad \text{for } i \in \{q, a, r\} \tag{S2}$$

$$\dot{b_i} = k_i^+ m_i r - k_i^- b_i - \frac{\epsilon(t^c)}{n_i} b_i - \lambda(\epsilon, B) \cdot b_i \quad \text{for } i \in \{q, a, r\} \tag{S3}$$

$$\dot{p_i} = \frac{\epsilon(t^c)}{n_i} b_i - \lambda(\epsilon, B) \cdot p_i \quad \text{for } i \in \{q, a\} \tag{S4}$$

$$\dot{r} = \frac{\epsilon(t^c)}{n_r} b_r - \lambda(\epsilon, B) \cdot r + \sum_{j \in \{q,a,r\}} \left((\frac{\epsilon(t^c)}{n_j} + k_j^-) b_j - k_j^+ m_j r \right) \tag{S5}$$

$$\dot{t^c} = \nu(t^u, \sigma) \cdot p_a - \epsilon(t^c) \cdot B - \lambda(\epsilon, B) \cdot t^c \tag{S6}$$

$$\dot{t^u} = \psi(T) \cdot \lambda(\epsilon, B) - \nu(t^u, \sigma) \cdot p_a + \epsilon(t^c) \cdot B - \lambda(\epsilon, B) \cdot t^u \tag{S7}$$

(Model I)

Supplementary Table S1 displays the values, as well as the physiological meaning, of all the parameters used in (Model I) and defining the rates of the reactions listed above. The formulae for the regulation functions and reaction rates, as well as their significance,

are given in Supplementary Table S2. Notably, the expression for λ enforces the constant cell mass trade-off identified by Weisse et al. [2]. Since the total mass of protein in the cell is by definition $M = n_r R + \sum_{j\in\{q,a\}} n_j p_j$, the mass of protein in the cell is always constant because:

$$
\begin{aligned}
\dot{M} = n_r \dot{R} + \sum_{j\in\{q,a\}} n_j \dot{p_j} \Leftrightarrow \dot{M} = n_r \sum_{j\in\{q,a\}} \dot{b_j} + n_r \dot{r} + \sum_{j\in\{q,a\}} n_j \dot{p_j} \Leftrightarrow \\
\Leftrightarrow \dot{M} = \sum_{j\in\{q,a,r\}} n_j \left(\frac{\epsilon}{n_j} b_j\right) - \lambda \left(n_r R + \sum_{j\in\{q,a\}} n_j p_j\right) \Leftrightarrow \\
\Leftrightarrow \dot{M} = \epsilon B - \lambda M \Leftrightarrow \\
\Leftrightarrow \dot{M} = \epsilon B - \frac{\epsilon B}{M} \cdot M = 0
\end{aligned}
\tag{S8}
$$

Supplementary Table S1: **Parameters appearing in the cell model ODEs and Supplementary Table S2.**

Param.	Description	Value	Units*	Source
M	Total cell mass (in amino acids)	$1.19 \cdot 10^9$	$aa^{\#}$	[3]
σ	Extracellular nutrient quality	From 0 to 1	None	–
$\bar{\phi}_q$	Housekeeping prot. mass fraction§	0.59	None	[4]
**Reaction rates**				
ϵ_{max}	Max. translation elongation rate	72,000	$aa^{\#}/h$	[5]
ν_{max}	Max. tRNA charging rate	$4,046.9$	h^{-1}	Fitted
ψ_{max}	Max. tRNA synthesis per unit growth rate	$4.32 \cdot 10^5$	nM	[6]ℶ
K_ϵ	Michaelis constant for translation elongation	$1,239.7$	nM	Fitted
K_ν	Michaelis constant for tRNA charging	$1,239.7$	nM	Fitted
τ	Michaelis constant for ppGpp signaling (determines ψ and F_r)	1	None	[6]
**Gene expression**				
c_i	Concentration of gene i DNA‡	1	nM	Convention
α_q	Promoter strengthℵ for gene q	Unspecified§	None	–
α_a	Promoter strengthℵ for gene a	$3.945 \cdot 10^5$	None	Fitted
α_r	Promoter strengthℵ for gene r	$4.070 \cdot 10^5$	None	Fitted
β_i	mRNA degradation rate‡	6	h^{-1}	[2]
k_i^+	mRNA-ribosome binding rate‡	60	$\frac{1}{nM \cdot h}$	[2]
k_i^-	mRNA-ribosome dissociation rate‡	60	h^{-1}	[2]
n_q	Number of amino acids in protein p_q	300	$aa^{\#}/nM$	[2]
n_a	Number of amino acids in protein p_a	300	$aa^{\#}/nM$	[2]
n_r	Number of amino acids in rib. protein	7,459	$aa^{\#}/nM$	[2]

**E. coli* volume is $\approx 10^{-18}$ m^3, so 1 nM is roughly equivalent to 1 molecule/cell [7].
#Amino acid residues. ‡ Identical across all native genes $i \in \{q, a, r\}$.
§The final simplified (Model III) avoids explicitly modeling housekeeping genes.
ℵPromoter strength measured as the maximum mRNA synthesis rate per gene copy divided by the cell growth rate at time of measurement.
ℶIn [6], maximum tRNA synthesis rates of $1.08 \cdot 10^6$ nM/h are said to be achieved in very rich media, in which the growth rate is equal to ≈ 2.5 h^{-1}, hence us dividing the former value by the latter.

Supplementary Table S2: **Functions and notations appearing in the host cell model ODEs.**

Func./Not.	Description	Formula	Units*
B	Number of translating ribosomes	$\sum_{j\in\{q,a,r\}} b_j$	nM
T	Proxy for ppGpp concentration (inversely proportional to the alarmone's level)	t^c/t^u	None
	**Reaction rates**		
ϵ	Translation elongation rate	$\epsilon_{max} \cdot \frac{t^c}{t^c+K_\epsilon}$	$aa^{\#}/h$
λ	Growth/dilution rate	$\frac{\epsilon B}{M}$	h^{-1}
ψ	tRNA synthesis rate	$\psi_{max} \cdot \frac{T}{T+\tau}$	nM/h
ν	tRNA charging rate	$\nu_{max} \cdot \sigma \cdot \frac{t^u}{t^u+K_\nu}$	h^{-1}
	**Transcription regulation functions**		
F_q	Transcription regulation for housekeeping genes	Unspecified§	None
F_a	Transcription regulation for metabolic genes (constitutive)	1	None
F_r	Transcription regulation for ribosomal genes (via ppGpp)	$\frac{T}{T+\tau}$	None

***E. coli* volume is $\approx 10^{-18}$ m^3, so 1 nM is roughly equivalent to 1 molecule/cell [7].
#Amino acid residues.
§Defining F_q is not necessary because the final, simplified (Model III) avoids explicitly modeling housekeeping gene expression, as shown in Supplementary Note S1.5.

S1.2 The quasi-steady-state approximation

We now apply the QSS approximation to (Model I) by appreciating that mRNA-ribosome binding and unbinding occur on a much faster timescale than other processes in the cell [7]. Therefore, the mRNA-ribosome complex concentrations can be assumed to be in quasi-steady-state. Hence, for all $i \in \{q, a, r\}$ we have:

$$\dot{b_i} = 0 \Leftrightarrow k_i^+ m_i r - k_i^- b_i - \frac{\epsilon(t^c)}{n_i} b_i - \lambda(\epsilon, B) \cdot b_i = 0 \Leftrightarrow$$
$$\Leftrightarrow b_i = m_i r \cdot \left(\frac{k_i^- + \epsilon(t^c)/n_i + \lambda(\epsilon, B)}{k_i^+} \right)^{-1} = \frac{m_i}{k_i(\epsilon, B)} \cdot r \tag{S9}$$

S1.3 Neglecting the mRNA-Ribosome complex dilution

The retrieval of the mRNA-ribosome complexes' concentrations using Equation (S9) is nevertheless hindered by the dilution term $\lambda(\epsilon, B)$ in $k_i(\epsilon, B)$, which itself depends on the total concentration of translating ribosomes. Instead of the full expression for $k_i(\epsilon, B)$, it would therefore be desirable to use an approximation given by Equation (S10), which neglects dilution due to cell growth and thus always slightly underestimates k_i.

$$k_i(\epsilon, B) \approx k_i(\epsilon) = \frac{k_i^- + \epsilon/n_i}{k_i^+} \tag{S10}$$

Let us now show that in all realistic scenarios this approximation is reasonably close to the actual value of $k_i(\epsilon, B)$. First, we substitute the formula for λ from Supplementary Table S2 into the full expression for $k_i(\epsilon, B)$ from Equation (S9), obtaining:

$$k_i(\epsilon, B) = \frac{k_i^- + \frac{\epsilon(t^c)}{n_i} + \lambda(\epsilon, B)}{k_i^+} = \frac{k_i^- + \frac{\epsilon(t^c)}{n_i} + \frac{\epsilon B}{M}}{k_i^+} = \frac{k_i^-}{k_i^+} + \frac{\epsilon}{k_i^+}\left(\frac{1}{n_i} + \frac{B}{M}\right) \tag{S11}$$

The effect of growth on this value is thus restricted to the $^B/_M$ component of the ϵ-dependent term of the expression. Now, let us consider the mass fraction of all ribosomal proteins in the cell $\phi_r = \frac{n_r R}{M}$. In the extensive experimental studies of *E. coli* growth conducted up to this point, it has not been found to exceed 0.3 [6]. Therefore, noticing that $B \leq R$ by definition, for all realistic purposes we can impose a cap on $^B/_M$:

$$\frac{B}{M} \leq \frac{R}{M} = \frac{\phi_r}{n_r} \leq \frac{0.3}{n_r} \tag{S12}$$

The metabolic and housekeeping proteins are assumed to span $n_q = n_a = 300$ amino acids, while a single ribosome comprises $n_r = 7549$ residues (see Supplementary Table S1). For the former two species we thus have

$$\frac{1}{n_q} = \frac{1}{n_a} = \frac{1}{300} \approx 3.33 \times 10^{-3} \gg 4.02 \times 10^{-5} = \frac{0.3}{7459} = \frac{0.3}{n_r} \geq \frac{B}{M} \tag{S13}$$

which means that the contribution of dilution to the ϵ-dependent term can be safely disregarded, vindicating the use of an approximation from Equation (S10).

Nonetheless, the situation is more complicated in the case of ribosomes, as for $n_i = n_r$ the $^B/_M$ component can reach up to 30% of $^1/_{n_r}$. However, for such a high number of amino acid residues, the whole ϵ-dependent term contributes very little to k_r, even at the highest translation rates possible. This can be demonstrated by calculating the approximation error $\delta_r(\epsilon)$ – that is, the difference between the estimate $k_r(\epsilon)$ and the real value $k_r(\epsilon, B)$, divided by the real value. Since the approximate value of k_r is always lower than the real one, dividing the difference by $k_r(\epsilon)$ instead of $k_r(\epsilon, B)$ bounds the approximation error from above:

$$\delta_r(\epsilon) = \frac{k_r(\epsilon, B) - k_r(\epsilon)}{k_r(\epsilon, B)} \leq \frac{k_r(\epsilon, B) - k_r(\epsilon)}{k_r(\epsilon)} = \frac{\frac{\epsilon}{k_r^+} \cdot \frac{0.3}{n_r}}{\frac{k_r^-}{k_r^+} + \frac{\epsilon}{k_r^+ n_r}} = \frac{0.3\epsilon}{k_r^- n_r + \epsilon} = \delta_r^{bnd}(\epsilon) \tag{S14}$$

It is easy to see that for positive translation elongation rates this bound increases monotonously with ϵ, so to cap the error for all cases it is enough to evaluate $\delta_r^{bnd}(\epsilon)$ for the maximum possible translation elongation rate. Using the value from Supplementary Table S1, we find this cap to be merely

$$\delta_r^{bnd}(\epsilon_{max}) = \frac{0.3\epsilon_{max}}{k_r^- n_r + \epsilon_{max}} = 4.15\% \tag{S15}$$

In summary, we have shown that for any transcript, it is possible to reliably approximate its lumped "mRNA-ribosome dissociation constant" $k_i(\epsilon, B)$ by an expression $k_i(\epsilon)$ that does not acknowledge the effects of dilution caused by cell growth, removing this value's dependence on the concentration of translating ribosomes (Equation (S11)). As shown in the next section, this allows to greatly simplify the analysis of competitive ribosome binding by different mRNAs. From this point on, we will be denoting the approximate value $k_i(\epsilon)$ simply as k_i to avoid clutter. The number of ribosomes translating

gene i's mRNA can therefore be denoted as

$$b_i \approx \frac{m_i}{k_i} r \tag{S16}$$

S1.4 Cell model with the QSS approximation

Let us now remember the definition for the total number of ribosomes in the cell R from Equation (S1). Together with Equation (S16), it yields

$$R = \sum_{\forall i} b_i + r = r(1 + \sum_{\forall i} \frac{m_i}{k_i}) \Leftrightarrow r = R/(1 + \sum_{\forall i} \frac{m_i}{k_i}) \Rightarrow$$
$$\Rightarrow b_i = \frac{m_i/k_i}{1 + \sum_{j \in \{q,a,r\}} m_j/k_j} R = \frac{m_i/k_i}{D} R \tag{S17}$$

where

$$D = 1 + \sum_{j \in \{q,a,r\}} m_j/k_j \tag{S18}$$

Consequently, we can also state that

$$B = \sum_{\forall i} b_i = \frac{\sum_{j \in \{q,a,r\}} m_j/k_j}{1 + \sum_{j \in \{q,a,r\}} m_j/k_j} R = (1 - \frac{1}{D}) R \tag{S19}$$

By providing the expressions for $\{b_i\}$ and B, Equations (S17) and (S19) rid us of the necessity to explicitly consider mRNA-ribosome binding and treat free and translating ribosomes as separate species. Moreover, with the complex dilution considered insignificant, the sum of Equation (S2)'s last three terms is roughly equal to $-\dot{b_i}$, which is zero due to the QSS assumption. This yields us a simplified (Model II). Importantly, Equation (S22) now gives an ODE for the total number of ribosomes R and not just the free ribosome count r.

$$\dot{m_i} = F_i c_i \alpha_i \lambda(\epsilon, B) - (\beta_i + \lambda(\epsilon, B)) m_i \qquad \text{for } i \in \{q,a,r\} \tag{S20}$$

$$\dot{p_i} = \frac{\epsilon(t^c)}{n_i} \cdot \frac{m_i/k_i}{1 + \sum_{j \in \{q,a,r\}} m_j/k_j} R - \lambda(\epsilon, B) \cdot p_i \qquad \text{for } i \in \{q,a\} \tag{S21}$$

$$\dot{R} = \frac{\epsilon(t^c)}{n_r} b_r - \lambda(\epsilon, B) \cdot R \tag{S22}$$

$$\dot{t^c} = \nu(t^u, \sigma) \cdot p_a - \epsilon(t^c) \cdot B - \lambda(\epsilon, B) \cdot t^c \tag{S23}$$

$$\dot{t^u} = \psi(T) \cdot \lambda(\epsilon, B) - \nu(t^u, \sigma) \cdot p_a + \epsilon(t^c) \cdot B - \lambda(\epsilon, B) \cdot t^u \tag{S24}$$

(Model II)

If the system is in steady state (which here is indicated by drawing bars over the variables), a useful expression for a given gene's equilibrium protein concentration $\overline{p}_i$ can be derived as shown below.

$$\dot{p_i} = 0 \Leftrightarrow \frac{\overline{\epsilon}}{n_i} \cdot \frac{\overline{m}_i/\overline{k}_i}{\overline{D}} \overline{R} - \overline{\lambda}\overline{p}_i = 0 \Leftrightarrow$$
$$\Leftrightarrow \overline{p}_i = \frac{M}{n_i} \cdot \frac{\overline{m}_i/\overline{k}_i}{\sum_{j \in \{q,a,r\}} \overline{m}_j/\overline{k}_j} \tag{S25}$$

Importantly, Equation (S25) is merely a relation between the steady-state mRNA and protein concentrations, and does not allow to analytically determine $\overline{p}_i$ straightaway. This is because the formulae for k_i values include the translation elongation rate ϵ, while the steady-state mRNA concentrations $\overline{m}_i$ depend on the cell growth rate λ. Since ϵ and λ are not constant and depend on the cell's state, the values of $\overline{m}_i$ and $\overline{k}_i$ cannot be found based on Equation (S25) alone.

Let us conclude this section by stating a useful relation for $\overline{\phi}_i$, the steady-state mass fraction of protein p_i. Multiplying the protein's concentration by one molecule's weight in amino acid residues and dividing it by the cell's total protein mass, we get:

$$\overline{\phi}_i = \frac{n_i \overline{p}_i}{M} = \frac{\overline{m}_i/\overline{k}_i}{\sum_{j \in \{q,a,r\}} \overline{m}_j/\overline{k}_j} \tag{S26}$$

S1.5 Neglecting the housekeeping genes

The key property of housekeeping genes is that their expression remains constant and growth rate-independent. In fact, the assumption that ϕ_q is unchanging regardless of the growth rate or nutrient availability has been shown to hold under a wide range of conditions [4], yielding reliable predictions about the dynamics of cellular processes and not just their steady states [6]. Therefore, instead of explicitly modeling transcription and translation of the housekeeping genes, we can further simplify the model by assuming

that $\phi_q \equiv \overline{\phi}_q \equiv 0.59$ (and thus $p_q \equiv \overline{p}_q \equiv \frac{\overline{\phi}_q M}{n_q}$) in all cases [4]. Therefore, at any point in time

$$\frac{m_q/k_q}{\sum_{j\in\{q,a,r\}} m_j/k_j} = \overline{\phi}_q \tag{S27}$$

This in turn means that

$$\sum_{j\in\{q,a,r\}} m_j/k_j = \frac{1}{1-\overline{\phi}_q} \sum_{j\in\{a,r\}} m_j/k_j \tag{S28}$$

and

$$D = 1 + \frac{1}{1-\overline{\phi}_q} \sum_{j\in\{a,r\}} m_j/k_j \tag{S29}$$

B, the total concentration of mRNA-ribosome complexes, including those with the housekeeping gene mRNA, can still be found from D as described by Equation (S19). Consequently, it is possible to model ribosomal and metabolic protein expression, cell growth, and tRNA charging and synthesis without knowing the exact mRNA concentrations for the housekeeping genes. Hence, we can modify (Model II) to obtain (Model III). This is the simplified mechanistic cell modeling framework that we describe in the article's main text (Figure 2 and Equations (15)-(20) in Methods) and employ to obtain the results presented in this paper.

$$\dot{m_i} = F_i c_i \alpha_i \lambda(\epsilon, B) - (\beta_i + \lambda(\epsilon, B)) m_i \qquad \text{for } i \in \{a, r\} \tag{S30}$$

$$\dot{p_a} = \frac{\epsilon(t^c)}{n_a} \cdot \frac{m_a/k_a}{1 + \frac{1}{1-\overline{\phi}_q}\sum_{j\in\{a,r\}} m_j/k_j} R - \lambda(\epsilon, B) \cdot p_a \tag{S31}$$

$$\dot{R} = \frac{\epsilon(t^c)}{n_r} \cdot \frac{m_r/k_r}{1 + \frac{1}{1-\overline{\phi}_q}\sum_{j\in\{a,r\}} m_j/k_j} R - \lambda(\epsilon, B) \cdot R \tag{S32}$$

$$\dot{t^c} = \nu(t^u, \sigma) \cdot p_a - \epsilon(t^c) \cdot B - \lambda(\epsilon, B) \cdot t^c \tag{S33}$$

$$\dot{t^u} = \psi(T) \cdot \lambda(\epsilon, B) - \nu(t^u, \sigma) \cdot p_a + \epsilon(t^c) \cdot B - \lambda(\epsilon, B) \cdot t^u \tag{S34}$$

(Model III)

Using Equation (S28), it is also possible to rewrite Equations (S25) and (S26) to obtain

expressions for the steady-state protein concentrations and mass fractions:

$$\overline{p}_i = \frac{M}{n_i} \cdot \frac{\overline{m}_i/\overline{k}_i}{\frac{1}{1-\overline{\phi}_q} \sum_{j\in\{a,r\}} \overline{m}_j/\overline{k}_j} \tag{S35}$$

$$\overline{\phi}_i = \frac{\overline{m}_i/\overline{k}_i}{\frac{1}{1-\overline{\phi}_q} \sum_{j\in\{a,r\}} \overline{m}_j/\overline{k}_j} \tag{S36}$$

S1.6 Nutrient upshift modeling

In this section, we outline a simple extension to our model that allows to simulate the dynamic shifts in bacterial resource allocation in a nutrient upshift, i.e., a sharp improvement in the culture medium's nutrient quality due to an additional nutrient being supplemented to it.

The medium's nutrient quality in our model is given by the coefficient $0 < \sigma \leq 1$. A straightforward way to model nutrient upshift is to increase this factor from a lower pre-shift value σ_{pre} to a higher post-shift value σ_{post}. This, however, fails to capture that different metabolic pathways, and thus different metabolic proteins, are needed to import different nutrients and convert them into protein synthesis precursors. In absence of a certain nutrient, the corresponding enzyme is functionally useless and expressing it only burdens the cell, so the bacterium does not express the unneeded protein so as to optimize its growth rate. When the nutrient in question is sensed by the cell, the matching enzyme's expression is upregulated in response; however, some time may pass until the new high-expression equilibrium concentration of the metabolic enzyme is achieved [8, 9].

When considering a nutrient upshift, let us then use $\tilde{a}$ to denote the gene responsible for the rate-limiting step in the metabolism of the newly introduced nutrient. Notably, since we are considering a single gene among plenty, any changes in the regulation of its expression do not invalidate our general assumption that, from a coarse-grained point of view, the metabolic genes are expressed constitutively. In line with the general form of ODEs that we use for gene expression, we describe its mRNA and protein concentrations' dynamics using Equations (S37)-(S38), with all parameters having the same meaning as for the metabolic genes in general (i.e., $\alpha_{\tilde{a}}$ is its promoter strength, $n_{\tilde{a}}$ is the protein's length in amino acids, etc.). Besides the promoter strength $\alpha_{\tilde{a}}$, which is smaller than α_a to capture the fact that the rate-limiting enzyme is only one among many metabolic

genes, all parameters of the gene $\tilde{a}$ are assumed equal to those for the metabolic protein class a – that is, $c_{\tilde{a}} = c_a$, $\beta_{\tilde{a}} = \beta_a$, $n_{\tilde{a}} = n_a$, etc..

$$\dot{m}_{\tilde{a}} = F_{\tilde{a}} c_{\tilde{a}} \alpha_{\tilde{a}} \lambda(\epsilon, B) - (\beta_{\tilde{a}} + \lambda(\epsilon, B)) m_i \tag{S37}$$

$$\dot{p}_{\tilde{a}} = \frac{\epsilon(t^c)}{n_{\tilde{a}}} \cdot \frac{m_{\tilde{a}}/k_{\tilde{a}}}{1 + \frac{1}{1-\phi_q} \sum_{j \in \{a,r\}} m_j/k_j} R - \lambda(\epsilon, B) \cdot p_{\tilde{a}} \tag{S38}$$

As for the transcription regulation function $F_{\tilde{a}}$, the transcription of metabolic enzyme genes it is known to turn on almost momentarily upon nutrient upshifts [8], so we assume that just after the upshift we have it at its maximum value $F_{\tilde{a}} = 1$. Owing to their short half-lives, for the sake of simplicity we also assume that both the specific metabolic gene's mRNA concentration $m_{\tilde{a}}$ and the overall metabolic mRNA concentration m_a are in quasi-steady-state relative to the slower changes in the metabolic protein's concentrations [7]. Then, ever since the upshift occurs, the mRNA concentrations are given by

$$m_{\tilde{a}} = \frac{F_{\tilde{a}} c_{\tilde{a}} \alpha_{\tilde{a}} \lambda}{\beta_{\tilde{a}} + \lambda} = \frac{1 \cdot c_{\tilde{a}} \alpha_{\tilde{a}} \lambda}{\beta_{\tilde{a}} + \lambda} \tag{S39}$$

$$m_a = \frac{1 \cdot c_a \alpha_a \lambda}{\beta_a + \lambda} \tag{S40}$$

and therefore

$$\frac{m_{\tilde{a}}}{m_a} = \frac{\alpha_{\tilde{a}}}{\alpha_a} = \overline{\Phi} = const \tag{S41}$$

Now, consider the mole fraction of enzymes of interest among all metabolic proteins:

$$\Phi = \frac{p_{\tilde{a}}}{p_a} \tag{S42}$$

Unlike $\overline{\Phi}$, the value Φ is non-constant. However, together these two notations allow us to compactly rewrite Equation (S38) as

$$\dot{p}_{\tilde{a}} = \overline{\Phi} \dot{p}_a + \lambda p_a \cdot (\overline{\Phi} - \Phi) \tag{S43}$$

Applying the chain rule to differentiation to Equation (S43) and Equation (S31), we find that the protein mole fraction then evolves according to Equation (S44), which also

shows that we have $\Phi = \overline{\Phi}$ when the cell reaches its new post-shift steady state.

$$\dot{\Phi} = (\overline{\Phi} - \Phi) \cdot \frac{\epsilon}{n_a} \cdot \frac{m_a/k_a}{1 + \frac{1}{1-\phi_q} \sum_{j \in \{a,r\}} m_j/k_j} \cdot \frac{R}{p_a} \tag{S44}$$

With this in mind, let us go back to the definition of the tRNA aminoacylation rate from Supplementary Table S2 and apply it to the upshift scenario. The already-present pre-shift carbon source, with nutrient quality σ_{pre}, still contributes to aminoacyl-tRNA synthesis. However, protein precursor production from the newly added nutrient is also present, so we sum the two quantities:

$$\nu = \nu_{max} \cdot \sigma_{pre} \cdot \frac{t^u}{t^u + K_\nu} \cdot p_a + \nu_{max} \cdot \Delta\sigma \cdot \frac{t^u}{t^u + K_\nu} \cdot p_a = \nu_{max} \cdot (\sigma_{pre} + \Delta\sigma) \cdot \frac{t^u}{t^u + K_\nu} \cdot p_a \tag{S45}$$

Here, the new nutrient's quality $\Delta\sigma$ captures the number of aminoacyl-tRNAs that a single nutrient molecule can produce at a given time. This value is dependent on the concentration of the protein that limits the rate of the nutrient's metabolization. Denoting the coefficient of this proportionality as $\tilde{\sigma}$, we get:

$$\Delta\sigma = \tilde{\sigma} p_{\tilde{a}} = \tilde{\sigma} \Phi p_a \tag{S46}$$

We also know that in the steady state, the overall post-shift tRNA aminoacylation rate is given by Equation (S47).

$$\nu = \nu_{max} \cdot \sigma_{post} \cdot \frac{t^u}{t^u + K_\nu} \cdot p_a \tag{S47}$$

Together, Equations (S46), (S45), and (S47) allow to define the tRNA aminoacylation rate during nutrient upshifts in terms of the rate-limiting enzyme's molar fraction as

$$\nu(t^u, \Phi) = \nu_{max} \cdot \left(\sigma_{pre} + (\sigma_{post} - \sigma_{pre}) \cdot \frac{\Phi}{\overline{\Phi}} \right) \cdot \frac{t^u}{t^u + K_\nu} \cdot p_a \tag{S48}$$

Therefore, the cell's state during nutrient upshifts can be simulated simply by keeping all of the original (Model III) ODEs, augmenting the model with Equation (S44) for Φ, and replacing the formula for the tRNA aminoacylation rate from Supplementary Table S2 with the one in Equation (S48). As for the initial condition for the additional variable Φ right after the new nutrient is added, it depends on the level of $p_{\tilde{a}}$ before the upshift.

This is likely to be nutrient- and enzyme-specific, but is usually not zero, as in most cases the cell growth rate experiences a sharp initial rise right after the introduction of a new nutrient, which means that to some extent it can metabolize the new carbon source straightaway [9]. Here, we use a crude estimate based on the ratio of steady-state growth rates in preshift and postshift media:

$$\Phi_{init} = \frac{\overline{\lambda}_{post}}{\overline{\lambda}_{pre}} \tag{S49}$$

As a showcase of our model predicting nutrient upshift dynamics, we plot its forecasts alongside the experimental data for the addition of different nutrient to a medium with succinate as the only carbon source [8]. Given that the steady-state growth rates in all media were known, we estimated the corresponding pre- and post-shift σ values by going through $1,000$ evenly spaced points on the interval $0.01 \leq \sigma \leq 1$ and finding the one that yields the closest steady-state growth rate to the one recorded for a given combination of nutrients. As it can be seen in Supplementary Figure S1, our model's predictions indeed adequately match the nutrient upshift dynamics in almost all scenarios. An exception to this is the addition of xylose to the medium (green line). This can be attributed to our very rough estimate of an initial condition. Indeed, while the steady-state growth rate for the succinate medium with xylose is very close to that for the succinate medium with glycerol (0.73 vs 0.75, respectively), their corresponding experimentally observed upshift dynamics are very distinct. This illustrates the limitation of estimating initial enzyme levels based on steady-state growth rates alone, as we do in Equation (S49).

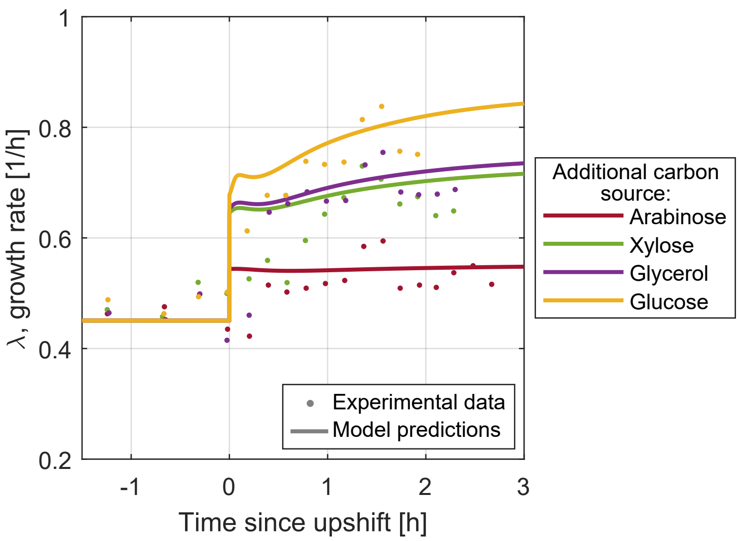

Supplementary Figure S1: **Cell growth over time in different nutrient upshift scenarios**. A nutrient upshifts constitutes the addition of different additional nutrients to a culture medium with succinate as the only carbon source. Dots stand for experimental measurements from [8], whereas lines show our model's predictions. The steady state growth rates with arabinose-, xylose-, glycerol-, and glucose-supplemented media are 0.55 h^{-1}, 0.73 h^{-1},0.75 h^{-1}, and 0.55 h^{-1} respectively. The steady-state growth rate in the pre-shift medium is 0.45 h^{-1} [8]. Source data are provided as a Source Data file.

S2 Parameter fitting

Here, we provide the details of our fitting procedure for the model parameters that were not taken from literature. First, we outline how the cell model was modified to incorporate the effects of the ribosome-inactivating antibiotic chloramphenicol, since it had been present in the culture media of some *E. coli* cells in the experiment whose results we used for fitting [10]. Next, we describe the parameters and outcome of the Markov Chain Monte Carlo (MCMC) simulation that we used to infer the parameter values from data. Finally, we show how the inferred values were postprocessed to make the model more realistic.

S2.1 Modeling ribosome inactivation

In order to determine the parameters' values, we fit our model to experimental data obtained by Scott et al. [10]. In their experiment, the growth rate and the ratio of total RNA mass to the overall mass of protein in the cell were measured for *E. coli* grown in different conditions (for our fitting, the RNA:protein ratios that they recorded were multiplied by a conversion factor of 0.4558 to obtain ribosomal mass fractions [6]). These sundry growth conditions were modeled by varying the culture medium's nutritional quality and the amount of chloramphenicol present in it. Chloramphenicol is an antibiotic that inactivates translating ribosomes by binding them instead of the tRNA that carries the amino acid supposed to be added to the peptide chain. We assume that the presence of chloramphenicol introduces two additional kinds of reactions besides those displayed in Supplementary Note S1.1:

- **Ribosome inactivation:** $b_i + h \xrightarrow{k_{cm}} B_{cm} \quad \text{for } i \in \{q, a, r\}$
- **Inactivated ribosome dilution:** $B_{cm} \xrightarrow{\lambda} \varnothing$

Here, b_i denotes the mRNA-ribosome complex for gene i, h signifies chloramphenicol, and B_{cm} the inactivated ribosome. The first reaction equation stands for ribosome inactivation by chloramphenicol, whereas the second describes the dilution of the inactivated ribosomes due to cell division.

Notably, we do not explicitly model the antibiotic's transport across the cell membrane, so h denotes the concentration of chloramphenicol in the medium. The value of the binding

rate constant k_{cm} therefore implicitly accounts for conversion between the extracellular and the intracellular antibiotic levels. Moreover, the share of chloramphenicol molecules bound by ribosomes is assumed to be very small compared to the antibiotic's abundance in the media, so we consider h constant.

Then, let $R = r + \sum_{j\in\{q,a,r\}} b_i$ denote exclusively non-inactivated ribosomes, and let B_{cm} signify the concentration of ribosomes bound by chloramphenicol. The experimentally measured ribosome fraction, nevertheless, still includes both operational and disabled ribosomes. Likewise to Supplementary Note S1.1, we obtain the unsimplified ODE (Model IV).

$$\dot{m_i} = F_i c_i \alpha_i \lambda(\epsilon, B) - (\beta_i + \lambda(\epsilon, B))m_i - k_i^+ m_i r + k_i^- b_i + \frac{\epsilon(t^c)}{n_i} b_i \qquad \text{for } i \in \{q, a, r\} \quad \text{(S50)}$$

$$\dot{b_i} = k_i^+ m_i r - k_i^- b_i - \frac{\epsilon(t^c)}{n_i} b_i - \lambda(\epsilon, B) \cdot b_i - k_{cm} h b_i \qquad \text{for } i \in \{q, a, r\} \quad \text{(S51)}$$

$$\dot{p_i} = \frac{\epsilon(t^c)}{n_i} b_i - \lambda(\epsilon, B) \cdot p_i \qquad \text{for } i \in \{q, a\} \quad \text{(S52)}$$

$$\dot{r} = \frac{\epsilon(t^c)}{n_r} b_r - \lambda(\epsilon, B) \cdot r + \sum_{j\in\{q,a,r\}} \left((\frac{\epsilon(t^c)}{n_j} + k_j^-) b_j - k_j^+ m_j r \right) \quad \text{(S53)}$$

$$\dot{t^c} = \nu(t^u, \sigma) \cdot p_a - \epsilon(t^c) \cdot B - \lambda(\epsilon, B) \cdot t^c \quad \text{(S54)}$$

$$\dot{t^u} = \psi(T) \cdot \lambda(\epsilon, B) - \nu(t^u, \sigma) \cdot p_a + \epsilon(t^c) \cdot B - \lambda(\epsilon, B) \cdot t^u \quad \text{(S55)}$$

$$\dot{B}_{cm} = k_{cm} h B - B_{cm} \lambda(\epsilon, B) \quad \text{(S56)}$$

(Model IV)

Applying the QSS approximation to Equation (S51), we obtain

$$b_i = m_i r \cdot \left(\frac{k_i^- + \epsilon(t^c)/n_i + \lambda(\epsilon, B) + k_{cm} h}{k_i^+} \right)^{-1} = \frac{m_i}{k_i(\epsilon, B, h)} r \quad \text{(S57)}$$

An additional non-negative term – that is, $k_{cm}h$ – can only render the growth-dependent component's contribution even less significant relative to other terms. Hence, the considerations outlined in Supplementary Note S1.3 remain valid and allow us to employ a growth-independent approximation for the mRNA-ribosome dissociation constant. We thus define $\tilde{k_i}$, the approximate mRNA-ribosome dissociation constant with adjustment

for chloramphenicol, as

$$\tilde{k}_i(\epsilon, h) = \frac{\epsilon/n_i + k_i^- + k_{cm}h}{k_i^+} \tag{S58}$$

Consequently, we can simplify (Model IV) analogously to how we reduced (Model I) in Supplementary Notes S1.4-S1.5, this time with $\tilde{k}_i$ instead of k_i. This yields us (Model V), which we used in our fitting procedure to predict a cell's behavior for given culturing conditions and parameter values.

$$\dot{m}_i = F_i c_i \alpha_i \lambda(\epsilon, B) - (\beta_i + \lambda(\epsilon, B)) m_i - k_{cm} h \cdot \frac{m_i/\tilde{k}_i}{1 + \frac{1}{1-\bar{\phi}_q} \sum_{j \in \{a,r\}} m_j/\tilde{k}_j} R \quad \text{for } i \in \{a, r\} \tag{S59}$$

$$\dot{p}_a = \frac{\epsilon(t^c)}{n_a} \cdot \frac{m_a/\tilde{k}_a}{1 + \frac{1}{1-\bar{\phi}_q} \sum_{j \in \{a,r\}} m_j/\tilde{k}_j} R - \lambda(\epsilon, B) \cdot p_a \tag{S60}$$

$$\dot{R} = \frac{\epsilon(t^c)}{n_r} \cdot \frac{m_r/\tilde{k}_r}{1 + \frac{1}{1-\bar{\phi}_q} \sum_{j \in \{a,r\}} m_j/\tilde{k}_j} R - \lambda(\epsilon, B) \cdot R - k_{cm} h B \tag{S61}$$

$$\dot{t^c} = \nu(t^u, \sigma) \cdot p_a - \epsilon(t^c) \cdot B - \lambda(\epsilon, B) \cdot t^c \tag{S62}$$

$$\dot{t^u} = \psi(T) \cdot \lambda(\epsilon, B) - \nu(t^u, \sigma) \cdot p_a + \epsilon(t^c) \cdot B - \lambda(\epsilon, B) \cdot t^u \tag{S63}$$

$$\dot{B}_{cm} = k_{cm} h B - \lambda(\epsilon, B) \cdot B_{cm} \tag{S64}$$

(Model V)

S2.2 MCMC fitting

Fitting was required to determine the values of:

- ν_{max}, the maximum tRNA charging rate
- α_a and α_r, the metabolic and ribosomal genes' promoter strengths
- K_ϵ and K_ν, the Michaelis constants determining the translation elongation and tRNA charging rates, respectively
- k_{cm}, the chloramphenicol binding rate constant

We performed the fitting with the help of the Differential Evolution Adaptive Metropolis (DREAM) algorithm [11], running 10 chains in parallel for 20,000 steps. As a Markov

Supplementary Table S3: **Parameters fitted to experimental data and the initial guesses for their values input to the DREAM algorithm.**

Parameter(s)	Description	Initial guess (prior mean)	Units	Source
$K_\epsilon = K_\nu$	Michaelis constants determining the translation elongation and tRNA charging rates	$3,000$	nM	[12]
k_{cm}	Chloramphenicol binding rate constant	$3.594 \cdot 10^{-4}$	$\frac{1}{nM \cdot h}$	[2]
$\alpha_r : \alpha_a$	Ribosomal to metabolic gene promoter strength ratio	1	None	Convention
ν_{max}	Maximum tRNA charging rate	$6,000$	h^{-1}	[6]

Chain Monte Carlo (MCMC) method, it constructs Markov chains whose states are the possible sets of parameter values and whose stationary distribution equals the probability distribution of these parameter sets. To this end, every step a new set of parameter values is selected according to some proposal distribution; then, the likelihoods of the experimental measurements are calculated; finally, the new parameter values are accepted with a probability that is proportional to the ratio of the two likelihoods.

In our case, the experimentally measured values of the steady-state growth rate $\overline{\lambda}$ and ribosomal mass fraction $\overline{\phi}_r$ were assumed to have independent normal distributions around the mean values predicted by the model with a given parameter set. We used the standard deviation values of 0.0447 h^{-1} and 0.018976 for $\overline{\lambda}$ and $\overline{\phi}_r$ respectively, calculated as the average measurement errors across all conditions observed by Scott et al. [10]. In line with [6], the conversion of the RNA:protein mass ratio into ribosomal mass fraction was achieved by multiplying the measurement by 0.4558. The likelihood of experimental measurements for a given parameter set, up to a constant scaling factor that cancels out when the ratio of likelihoods is calculated, was therefore equal to:

$$\exp\left(\sum_{i\in I}\left(\frac{\overline{\lambda}_i - \hat{\lambda}_i}{0.0447}\right)^2 + \sum_{i\in I}\left(\frac{\overline{\phi}_{r_i} - \hat{\phi}_{r_i}}{0.018976}\right)^2\right) \tag{S65}$$

In Equation (S65), I is the set of all culture conditions for which the measurements were taken and the model was simulated; $\overline{\lambda}_i$ and $\overline{\phi}_{r_i}$ are the steady-state growth rate and ribosomal mass fraction predicted by the model for the condition $i \in I$; $\hat{\lambda}_i$ and $\hat{\phi}_{r_i}$ are the experimentally observed values of these quantities.

Notably, the model was fitted solely to the datapoints with growth rates above 0.3 h^{-1}, because at slower growth rates metabolic regulation is predominated by mechanisms unconsidered by our model and experimental measurements can be unreliable (see the main text's Results section and [6]). Furthermore, to improve the fitting procedure's efficiency, we reduced the number of values to be determined as follows. First, near-optimal resource allocation is known to be achieved when K_ϵ and K_ν are roughly equal [6]. Consequently, we treated both Michaelis constants as a single parameter whose value was to be determined. Second, in our initial MCMC runs, we observed that the likelihood changes very little when the absolute values of α_a and α_r are varied but their ratio stays the same (Supplementary Figure S2b) . Therefore, instead of fitting both values, we fixed α at a crude order-of-magnitude estimate of $\alpha_a = 3.89 \cdot 10^5$, making the metabolic genes account for a third of all mRNA synthesis in the cell at a reference growth rate of 0.7 h^{-1}[3], and used MCMC to determine the α_r:α_a ratio. Considering different pairwise combinations of other fitted parameters does not show relationships as clear as that between α_a and α_r (Supplementary Figure S2e), hence them being fit independently.

Prior distributions before the MCMC run we assumed to be normal for all parameters, with the variance being equal to 0.25 of the mean; the admissible ranges for all parameters were assumed to be between $^1/_{50}$ and 50 times the prior distribution's mean. As for the prior mean values, for $\alpha_r : \alpha_a$ it was set at 1 as a convention. For all other parameters, the prior distributions' mean were initial guesses from published literature (Supplementary Table S3). While for $K_\nu = K_\epsilon$ and k_{cm} the values could be borrowed from the corresponding papers straightforwardly, the tRNA aminoacylation rate in [6] was calculated in the relative amioacyl-tRNA mass abundance units and as a function metabolic protein mass fraction in the cell. Conversely, in our model the tRNA aminoacylation rate is found from metabolic protein concentrations p_a and has the units of nM/h. Since 0.001 tRNA abundance units per hour in [6] is roughly equivalent to 10^6 nM/h and $\phi_a = \frac{n_a p_a}{M}$, the original value of 20 h^{-1} was transformed into

$$20 \cdot \frac{10^6}{0.001} \cdot \frac{n_a}{M} \approx 6,000\ h^{-1} \tag{S66}$$

After running the MCMC algorithm, the estimated parameter values were obtained by finding the mode of the inferred posterior probability distribution. We then used these values to improve upon our estimate for α_a as described in Supplementary Note S2.3.

In order to asses the fit's sloppiness, we estimated the posterior distribution's Fisher Information Matrix (FIM) by retrieving the pseudo-inverse of the variance-covariance matrix [2, 13]. The magnitudes of the FIM's eigenvalues were spread over almost 26 orders of magnitude (Supplementary Figure S2c), hinting that the model is truly sloppy, i.e., that its behavior is largely independent from the exact values of most parameters [14]. Indeed, as shown in Supplementary Figure S2d, the estimates of parameter sensitivities based on the FIM [13] reveal that the model is relatively insensitive to changes in most parameter values. An exception to this is the ratio between the metabolic and ribosomal genes' promoter strengths $\alpha_r : \alpha_a$, the sensitivity to which is overwhelmingly large when compared to all other parameters. This can likely be explained by the fact that under our model the cell achieves near-optimal growth rates by managing resource allocation between ribosome and metabolic gene synthesis. Hence, a very different regulatory response may be required from the cell if the transcription rates of the two genes' mRNAs are altered, so the cell's behavior in given conditions may significantly change. The FIM allows to estimate the model's local parameter sensitivity, i.e., the likelihood function's derivative with respect to a given parameter [13]. Meanwhile, a more global picture is painted by exploring the parameter space over two orders of magnitude in Supplementary Figure S2e. In line with sloppy behavior, the likelihood's peak at the fitted parameter values is not sharp – rather, its logarithm stays almost as high in the vicinity of the optimal pair of parameter values.

S2.3 Scaling the promoter strengths

During parameter fitting, we used a crude order-of-magnitude estimate of $\alpha_a = 3.89 \cdot 10^5$. However, with the ratio between α_a and α_r known, the gene transcription rates can be determined with greater accuracy. To this end, we consider the *E. coli* cell in steady state growing at a rate of $\overline{\lambda} \approx 0.7/h$, which stands for one cell division per hour. The total rate of mRNA production in these conditions was experimentally observed to be $A = 1.02 \cdot 10^6 \ nucleotides/min = 6.12 \cdot 10^7 \ nucleotides/h$ [3]. In the following section, we express this quantity in terms of α_a, hence becoming able to deduce the metabolic gene transcription rate.

According to the MCMC fit, the maximum ribosomal gene transcription rate α_r constitutes 1.0318 of α_a (we also found $K_\epsilon = K_\nu$ to be $1,239.79 \ nM$, ν_{max} to be $4,046.9 \ h^{-1}$,

and k_{cm} to be $3.5436 \cdot 10^{-4}$ $(nM \cdot h)^{-1}$). Therefore, knowing the value of the ribosomal gene transcription regulation function $\overline{F}_r$ would enable us to find the total ribosomal mRNA production rate as

$$\text{Total } m_r \text{ synthesis rate} = \overline{F}_r \cdot \overline{\lambda} \cdot \alpha_r = \overline{F}_r \cdot \overline{\lambda} \cdot 1.0318 \cdot \alpha_a \tag{S67}$$

As for the housekeeping genes, we assume the housekeeping and metabolic gene transcripts to have 1) similar degradation rates $\beta_q = \beta_a = 6\ h^{-1}$ and 2) similar mRNA-ribosome dissociation constants $k_q = k_a$ (due to having the same lengths $n_a = n_q = 300$). Then, by Equation (S26) we have

$$\frac{\overline{\phi}_q}{\overline{\phi}_a} = \frac{\overline{m}_q/\overline{k}_q}{\overline{m}_a/\overline{k}_a} = \frac{\left(\frac{\overline{F}_q c_q \alpha_q \overline{\lambda}}{\beta_q + \overline{\lambda}} \cdot \frac{1}{\overline{k}_q}\right)}{\left(\frac{c_a \alpha_a \overline{\lambda}}{\beta_a + \overline{\lambda}} \cdot \frac{1}{\overline{k}_a}\right)} = \frac{\overline{F}_q c_q \alpha_q}{c_a \alpha_a} \Leftrightarrow$$

$$\Leftrightarrow \text{Total } m_q \text{ synthesis rate} = \frac{\overline{\phi}_q}{\overline{\phi}_a} \cdot \overline{\lambda} \cdot \alpha_a = \frac{0.59}{\overline{\phi}_a} \cdot \overline{\lambda} \cdot \alpha_a \tag{S68}$$

Importantly, our model assumes that a transcript can be bound by one ribosome at a time. In reality, however, multiple ribosomes can translate the same mRNA molecule simultaneously. Likewise to the resource competition model proposed by Qian et al. [7], we implicitly address this discrepancy by the adjusting the mRNA synthesis rates. Namely, the minimum distance between translating ribosomes is estimated at ≈ 25 codons [15], so a single mRNA molecule that spans n_i codons can be bound by up to ${}^{n_i}/_{25}$ ribosomes. Hence, the apparent production rate of the mRNA is the actual transcription rate times ${}^{n_i}/_{25}$. In combination with our previous considerations, the real total mRNA production rate in the cell is the sum of

$$\text{Actual total } m_q \text{ synthesis rate} = \left(\frac{n_q}{25}\right)^{-1} \cdot \frac{0.59}{\overline{\phi}_a} \cdot \overline{\lambda} \cdot \alpha_a$$

$$\text{Actual total } m_r \text{ synthesis rate} = \left(\frac{n_r}{25}\right)^{-1} \cdot \overline{F}_r \cdot 1.0318 \cdot \overline{\lambda} \cdot \alpha_a$$

$$\text{Actual total } m_a \text{ synthesis rate} = \left(\frac{n_a}{25}\right)^{-1} \cdot \overline{\lambda} \cdot \alpha_a$$

Finally, considering that A is given in nucleotides per minute, the rate of production of a given mRNA m_i should also be multiplied by the number of nucleotides it comprises, i.e., $3n_i$. In summary, all these steps give rise to the following relationship:

$$A = \alpha_a \cdot 0.7 \cdot \left(75 \cdot \frac{0.59}{\overline{\phi}_a} + 75 \cdot \overline{F}_r \cdot 1.0318 + 75 \right) \tag{S69}$$

Since model predictions change little upon the scaling of α_a and α_r, we start by simulating the model for the fitted parameter values given in Supplementary Table S3, which allows us to numerically estimate $\overline{F}_r$ and $\overline{\phi}_a$. Using $\sigma = 0.17054$ as a nutrient quality coefficient that yields $\lambda = 0.6926\ h^{-1}$, we obtain

$$\overline{\phi}_a \approx 0.312 \text{ and } \overline{F}_r \approx 0.0875$$

And therefore the metabolic protein transcription rate is

$$\alpha_a = 3.945 \cdot 10^5$$

as specified in Supplementary Table S1. The maximum ribosomal gene transcription rate is therefore obtained by multiplying this value by 0.99811, hence

$$\alpha_r = 4.070 \cdot 10^5$$

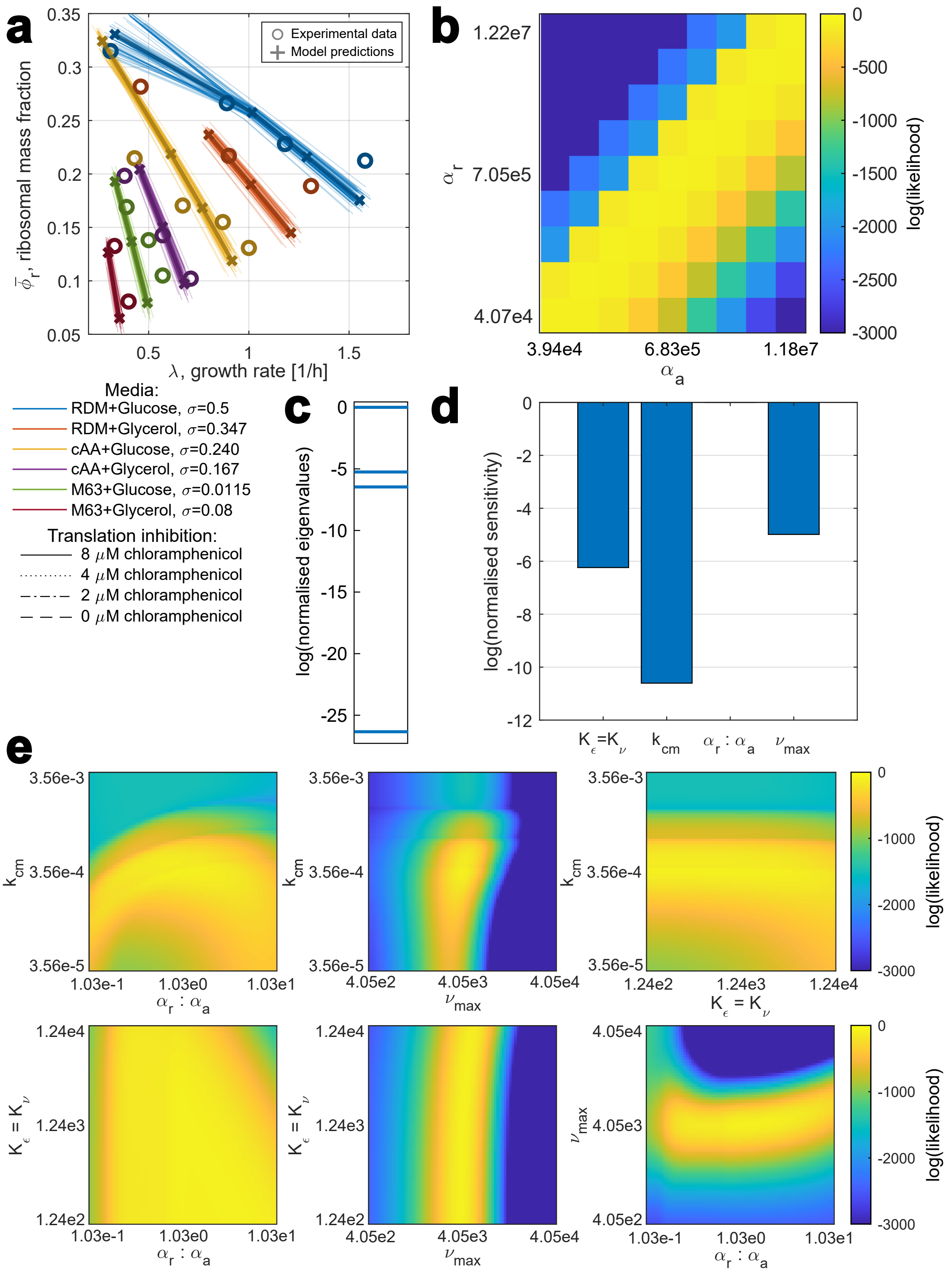
a
b
c
d
e
Experimental data
Model predictions
$\bar{\phi}_r$, ribosomal mass fraction
λ, growth rate [1/h]
Media:
RDM+Glucose, σ=0.5
RDM+Glycerol, σ=0.347
cAA+Glucose, σ=0.240
cAA+Glycerol, σ=0.167
M63+Glucose, σ=0.0115
M63+Glycerol, σ=0.08
Translation inhibition:
8 μM chloramphenicol
4 μM chloramphenicol
2 μM chloramphenicol
0 μM chloramphenicol
log(likelihood)
log(normalised eigenvalues)
log(normalised sensitivity)
$K_\epsilon = K_\nu$
k_{cm}
$\alpha_r : \alpha_a$
ν_{max}

Supplementary Figure S2: **MCMC fitting outcome and parameter sensitivity analysis.** **a** MCMC fitting outcome for the model. Thin lines: predictions for 100 parameter sets randomly drawn from the obtained probability distribution. Bold dark lines and crosses: model predictions for the maximum a posteriori estimate (found as the distribution's mode) of the parameter values, which we ultimately employ in our model and display in Supplementary Table S1. Bold circles: experimental measurements by Scott et al. [10] that were used in our fitting procedure. **b** Logarithms of experimental measurements' likelihood for different values of metabolic and ribosomal genes' promoter strengths α_a and α_r. Values of all other model parameters were taken from Supplementary Table S1. Observe how consistent the likelihoods stay along the diagonals going through the points that have the same $\alpha_r : \alpha_r$ ratios. **c** Logarithms of the FIM's eigenvalues normalized by the largest eigenvalue's magnitude. **d** Parameter sensitivities evaluated using the FIM, normalized to yield 1 when summed. The closer is the logarithm of parameter sensitivity to 0, the greater is the effect of the corresponding parameter's value on the model fit. **e** Logarithms of experimental measurements' likelihood for different pairwise combinations of parameter values. The axes for all parameters are logarithmic and range from 0.1 to 10 times the value determined by MCMC fitting. The pair of the fitted parameter values therefore always corresponds to the center of the heatmap. The color axis is the same for all subplots. Source data are provided as a Source Data file.

S3 Heterologous gene expression modeling

In this section, we describe how to extend the host cell model to include the expression of heterologous genes. Then, we derive the analytical relations between heterologous gene expression and the cell's state that we describe in the main text.

S3.1 Extending the mechanistic cell model

The simplified mechanistic (Model III), described in Supplementary Note S1.5, can easily be extended to model the expression of heterologous genes in the bacterium. In addition to the set of the modeled native genes $\{a, r\}$, let there also be a set of heterologous genes $X = \{x_1, x_2 \dots, x_L\}$. These genes are characterized by the same types of gene expression parameters as the native ones ($\{c_{x_l}\}, \{\alpha_{x_l}\}$, etc.) and each have a corresponding gene- and circuit-specific transcription regulation function F_{x_l}.

The existence of additional genes besides the native ones does not fundamentally change the considerations that enabled the reduction of the full mechanistic (Model I) to the simplified (Model III) outlined in Supplementary Notes S1.2-S1.5, so we can still apply the QSS approximation and avoid explicitly modeling housekeeping gene expression (the fixed housekeeping protein mass fraction can be still assumed to equal $\overline{\phi}_q = 0.59$ [4, 10, 16]). As a result, we use the same general form for the ODEs describing a gene's mRNA concentration m_i and protein concentration p_i as Equations (S30) and (S31) in (Model III). This lets us describe the host cell and the synthetic circuit by (Model VI).

$$\dot{m_i} = F_i c_i \alpha_i \lambda(\epsilon, B) - (\beta_i + \lambda(\epsilon, B)) m_i \qquad \text{for } i \in \{a, r\} \tag{S70}$$

$$\dot{p_a} = \frac{\epsilon(t^c)}{n_a} \cdot \frac{m_a/k_a}{1 + \frac{1}{1-\overline{\phi}_q} \sum_{j \in \{a,r\} \cup X} m_j/k_j} R - \lambda(\epsilon, B) \cdot p_a \tag{S71}$$

$$\dot{R} = \frac{\epsilon(t^c)}{n_r} \cdot \frac{m_r/k_r}{1 + \frac{1}{1-\overline{\phi}_q} \sum_{j \in \{a,r\} \cup X} m_j/k_j} R - \lambda(\epsilon, B) \cdot R \tag{S72}$$

$$\dot{t^c} = \nu(t^u, \sigma) \cdot p_a - \epsilon(t^c) \cdot B - \lambda(\epsilon, B) \cdot t^c \tag{S73}$$

$$\dot{t^u} = \psi(T) \cdot \lambda(\epsilon, B) - \nu(t^u, \sigma) \cdot p_a + \epsilon(t^c) \cdot B - \lambda(\epsilon, B) \cdot t^u \tag{S74}$$

$$\dot{m_{x_l}} = F_{x_l} c_{x_l} \alpha_{x_l} \lambda(\epsilon, B) - (\beta_{x_l} + \lambda(\epsilon, B)) m_{x_l} \qquad \text{for } x_l \in X \tag{S75}$$

$$\dot{p_{x_l}} = \frac{\epsilon(t^c)}{n_{x_l}} \cdot \frac{m_{x_l}/k_{x_l}}{1 + \frac{1}{1-\overline{\phi}_q} \sum_{j \in \{a,r\} \cup X} m_j/k_j} R - \lambda(\epsilon, B) \cdot p_{x_l} \qquad \text{for } x_l \in X \tag{S76}$$

(Model VI)

(Model VI) allows to numerically simulate the behavior of a cell expressing heterologous proteins, provided that the gene expression burden does not decrease the growth rate enough to activate the cell's stress response mechanisms, which our model does not consider (in the main text's Results section, we postulate a threshold of $\lambda = 0.3\ h^{-1}$, beyond which our model's predictions significantly diverge with experimental measurements). Moreover, by solving $\dot{p_i} = 0$, it can be seen that the formulae from Equations (S35)-(S36), which describe the steady-state protein mass fraction, still hold for both native and heterologous genes. However, they now include a heterologous protein component:

$$\bar{p}_i = \frac{M}{n_i} \cdot \frac{\overline{m}_i/\overline{k}_i}{\frac{1}{1-\bar{\phi}_q}\sum_{j\in\{a,r\}\cup X}\overline{m}_j/\overline{k}_j} \tag{S77}$$

$$\bar{\phi}_i = \frac{\overline{m}_i/\overline{k}_i}{\frac{1}{1-\bar{\phi}_q}\sum_{j\in\{a,r\}\cup X}\overline{m}_j/\overline{k}_j} \tag{S78}$$

We simulated (Model VI) for a generic heterologous gene, displaying the results in Figure 5 of the main text alongside the analytical predictions for gene expression, which we obtained using the relations derived in the following subsections of this section. We characterized the gene with the parameters given in Supplementary Table S4, explaining our choices in Supplementary Note S4.1. The same parameters describe the "gene of interest" *poi*, for which we show how to maximize its expression by a population of cells in Supplementary Note S3.7. Equations (S75)-(S76), which define sets of ODEs for all synthetic genes, in this case become a single pair of ODEs as the synthetic gene set has exactly one element, i.e., $X = \{x\}$ or $X = \{poi\}$.

Supplementary Table S4: **Parameters of the generic synthetic genes x and poi. The results of simulating their expression** are shown in Supplementary Figure S3 and the main text's Figure 5.

Parameter	Description	Value	Units
$c_x = c_{poi}$	Gene DNA copy number	Varied	nM
$\alpha_x = \alpha_{poi}$	Promoter strength	1,000	None
$\beta_x = \beta_{poi}$	mRNA degradation rate	6	h^{-1}
$k_x^+ = k_{poi}^+$	mRNA-ribosome binding rate	60	$\frac{1}{nM \cdot h}$
$k_x^- = k_{poi}^-$	mRNA-ribosome dissociation rate	60	h^{-1}
$n_x = n_{poi}$	Number of amino acids in protein p_i	300	aa/nM

S3.2 Modeling degradation of heterologous proteins

The half-life of most proteins is much longer than the cell doubling time [17] and the majority of the host cell's proteins are not actively degraded [18], which makes it possible to neglect active protein degradation and assume that protein are removed by dilution only. Nonetheless, the performance of some synthetic gene circuits may hinge on fast protein degradation. To enable the simulation of such designs, this section offers a means of accounting for active protein degradation by making several minor modifications to (Model VI). Since these adjustments nonetheless complicate the retrieval of the model's steady state, note that all of the analytical derivations in Supplementary Notes S3.4-S4.4.2 have been made for the case of negligible degradation rates for all proteins.

Suppose that all synthetic genes in the cell $\{x_1, x_2, \dots, x_L\}$ encode proteins that are actively degraded at rates $\{\delta_{x_1}, \delta_{x_1}, \dots, \delta_{x_L}\}$, where none, several, or all δ_{x_l} values may be zero. The ODEs for the synthetic protein concentrations must therefore include an additional term for protein degradation, turning Equation (S76) into

$$\dot{p_{x_l}} = \frac{\epsilon(t^c)}{n_{x_l}} \cdot \frac{m_{x_l}/k_{x_l}}{D} R - \lambda p_{x_l} - \delta_{x_l} p_{x_l} \tag{S79}$$

where D is the resource competition denominator that scales the translation rates of all proteins to account for competitive ribosome binding.

Due to the finite proteome cellular trade-off [2], the total protein mass in the cell M remains constant, which our model enforces through the definition of the growth rate λ. With protein in the cell now removed not only by dilution due to growth, but also by protein degradation, a new formula for λ can be obtained:

$$\dot{M} = 0 \Leftrightarrow n_r \dot{R} + \sum_{j \in \{q,a\} \cup X} n_j \dot{p_j} = 0 \Leftrightarrow n_r \sum_{j \in \{q,a\} \cup X} \dot{b_j} + n_r \dot{r} + \sum_{j \in \{q,a\} \cup X} n_j \dot{p_j} = 0 \Leftrightarrow$$
$$\Leftrightarrow \epsilon B - \lambda M - \sum_{x_l \in X} \delta_{x_l} n_{x_l} p_{x_l} = 0 \Leftrightarrow$$
$$\Leftrightarrow \lambda = \frac{\epsilon B}{M} - \frac{\sum_{x_l \in X} \delta_{x_l} n_{x_l} p_{x_l}}{M} \tag{S80}$$

As outlined in Supplementary Note S1.5, the fact that the mass fraction of native housekeeping proteins $\overline{\phi}_q$ in the cell remains constant regardless of the culture conditions

[4, 6] allows us to avoid explicitly considering the expression of this gene class, capturing it instead in the definition for D in Equation (S29). However, the derivations to obtain it hinged on the original formula for the cell growth rate. By substituting the new definition of λ into $\dot{\phi}_q = 0$, the updated formula for D – that is, Equation (S82) – can be obtained as follows:

$$\dot{\phi}_q = \frac{n_q}{M} \cdot \dot{p}_q = 0 \Leftrightarrow \frac{\epsilon(t^c)}{n_q} \cdot \frac{m_q/k_q}{\sum_{j\in\{q,a,r\}\cup X} m_j/k_j} \cdot R - \left(\frac{\epsilon B}{M} - \frac{\sum_{x_l\in X}\delta_{x_l} n_{x_l} p_{x_l}}{M}\right) p_q = 0 \Leftrightarrow$$

$$\Leftrightarrow \frac{m_q}{k_q} = \frac{\overline{\phi}_q\left(1-\frac{\sum_{x_l\in X}\delta_{x_l} n_{x_l} p_{x_l}}{\epsilon R}\right)\cdot \sum_{j\in\{a,r\}\cup X} m_j/k_j - \overline{\phi}_q \frac{\sum_{x_l\in X}\delta_{x_l} n_{x_l} p_{x_l}}{\epsilon R}}{1-\overline{\phi}_q\left(1-\frac{\sum_{x_l\in X}\delta_{x_l} n_{x_l} p_{x_l}}{\epsilon R}\right)} \tag{S81}$$

hence

$$D = 1 + \sum_{j\in\{q,a,r\}\cup X} m_j/k_j = 1 = m_q/k_q + \sum_{j\in\{a,r\}\cup X} m_j/k_j =$$
$$= 1 + \frac{\sum_{j\in\{a,r\}\cup X} m_j/k_j - \overline{\phi}_q \frac{\sum_{x_l\in X}\delta_{x_l} n_{x_l} p_{x_l}}{\epsilon R}}{1-\overline{\phi}_q\left(1-\frac{\sum_{x_l\in X}\delta_{x_l} n_{x_l} p_{x_l}}{\epsilon R}\right)} \tag{S82}$$

Notably, for $\delta_{x_l} = 0 \; \forall x_l \in X$ this definition is equivalent to the original Equation (S82) that neglects active degradation of proteins.

S3.3 Stochastic modeling of heterologous gene expression

Plugging the equations of (Model VI) into a numerical ODE integrator, such as the `ode15s` function used in our Matlab implementation (see the main text's Methods), allows to deterministically simulate the behavior of a cell expressing heterologous genes of choice while taking into account resource couplings and host-circuit interactions. However, synthetic gene circuits can also be significantly affected by noise [19], which calls for the ability to perform stochastic simulations of our model.

To this end, a hybrid modeling approach was chosen in the vein of prior studies [20]

– that is, the variables describing the host cell were assumed to be continuous and deterministic, whereas the variables describing the synthetic gene circuitry were considered discrete and stochastic. Indeed, our host cell model coarse-grains the host cell's genes into just a few classes and considers the overall pool of protein synthesis precursors as opposed to treating different tRNAs separately. The cell model's variables therefore capture the average behavior of multiple species represented by them, whose stochastic fluctuations can be expected to average out, vindicating our deterministic treatment of the system [20].

Therefore, the evolution of native protein mRNA and concentrations m_a, m_r, p_a, and R was simulated using Equations (S70)-(S72), with all the rates and variables calculated according to Supplementary Table S2. The charged and uncharged tRNA concentrations evolve according to the slightly amended Equations (S83)-(S84), in which the tRNA deaminoacylation flux, formerly defined as $\epsilon(t^c)\cdot B$, now excludes the translation of heterologous transcripts. Instead, the translation of the synthetic gene set $\{x_1,\ x_2, \dots\ x_L\} = X$ is treated as a stochastic reaction as we describe later in this section.

$$\dot{t^c} = \nu(t^u, \sigma) \cdot p_a - \epsilon(t^c) \cdot \left(B - R\frac{\sum_{x_l \in X} m_{x_l}/k_{x_l}}{D}\right) - \lambda(\epsilon, B) \cdot t^c \tag{S83}$$

$$\dot{t^u} = \psi(T) \cdot \lambda(\epsilon, B) - \nu(t^u, \sigma) \cdot p_a + \epsilon(t^c) \cdot \left(B - R\frac{\sum_{x_l \in X} m_{x_l}/k_{x_l}}{D}\right) - \lambda(\epsilon, B) \cdot t^u \tag{S84}$$

Meanwhile, for a heterologous gene x_l with mRNA and protein molecule counts of m_{x_l} and p_{x_l}, the following the stochastic reactions may occur, where all parameters have the same meaning as for the deterministic definition of the system.

- **Transcription** at rate $F_{x_l}\alpha_{x_l}c_{x_l}\lambda \cdot \left(\frac{n_{x_l}}{25}\right)^{-1}$. Increases m_{x_l} by $\frac{n_{x_l}}{25}$
- **mRNA degradation** at rate $\beta_{x_l}m_{x_l} \cdot \left(\frac{n_{x_l}}{25}\right)^{-1}$. Decreases m_{x_l} by $\frac{n_{x_l}}{25}$
- **mRNA dilution** at rate $\lambda m_{x_l} \cdot \left(\frac{n_{x_l}}{25}\right)^{-1}$. Decreases m_{x_l} by $\frac{n_{x_l}}{25}$
- **Translation** at rate $\frac{\epsilon}{n_{x_l}} \cdot \frac{m_{x_l}/k_{x_l}}{D} \cdot R$. Increases p_{x_l} by 1, decreases t^c by n_{x_l} (charged tRNAs being used up), increases t^u by n_{x_l} (uncharged tRNAs produced)
- **Protein dilution** at rate λp_{x_l}. Decreases p_{x_l} by 1

Sometimes, two RNA molecules can bind each other and mutually annihilate – for example, the *act* and *anti* gene transcripts in our RNA-based integral controller, which is described in the main text's Results and Supplementary Note S4.4. In this case, an additional stochastic annihilation reaction is introduced:

- **mRNA annihilation** at rate $\theta m_{act} m_{anti}$. Decreases m_{act} by $\frac{n_{act}}{25}$ and decreases m_{anti} by $\frac{n_{anti}}{25}$

Notably, the continuous part of the system is defined for molecule concentrations, while the stochastic reactions are defined for molecule counts. However, the volume of an *E. coli* cell is roughly equal to $10^{-18}\ m^3$. Hence, 1 nM is roughly equivalent to 1 molecule/cell [7], so we can use molecule counts and concentrations interchangeably. Hence, protein synthesis or dilution changes p_{x_l} by 1 while tRNA abundances change by n_{x_l} upon translation, as making one protein chain requires n_{x_l} amino acids. Nevertheless, mRNA molecules change by $\frac{n_{x_l}}{25}$ (rounded to the nearest integer) instead of 1 – this is because mRNA levels in our model are scaled to account for the possibility of a single transcript being simultaneously translated by multiple ribosomes, as discussed in Supplementary Note S2.3.

This definition of the continuous and stochastic parts of the hybrid model allows to simulate it on the time interval $t \in (t_{start},\ t_{finish})$ using a tau-leaping Algorithm S1, generally following the steps laid out by Hepp et al. [19]. Note that here all model variables are gathered into the vector $\mathbf{v} = (m_a\ m_r\ p_a\ R\ t^c\ t^u\ m_{x_1}\ \dots m_{x_L}\ p_{x_1} \dots p_{x_L})^\top$.

S3.4 Effect of heterologous gene expression on translation elongation and ribosomal gene transcription rates

The numerical simulations enabled by (Model VI) can be useful in validating gene circuit designs and ensuring desired responses despite the influence of resource competition and shifts in the growth rate. Nonetheless, the design of novel resource-aware controllers can also greatly benefit from analytical insights into the system's behavior. Although (Model VI) itself is not analytically tractable, some simplifying assumptions can provide an approximation of the intracellular variables' steady-state values, enabling analytical derivations. Specifically, we assume that the steady state values of the translation elongation rate $\overline{\epsilon}$ and the ppGpp-dependent ribosomal gene transcription regulation function $\overline{F}_r$

Algorithm S1 Hybrid tau-leaping cell model simulation algorithm.

Set $t = t_{start}$, $\mathbf{v} = \mathbf{v}_{start}$
while $t < t_{finish}$ **do**
  Find the deterministic change in variables $\Delta_{det}\mathbf{v}$ by integrating ODEs (S70)-(S72) and (S83)-(S84) over $(t,\ t + \Delta t)$
  Determine the number of times each stochastic reaction occurred over $(t,\ t + \Delta t)$ by sampling a Poisson distribution with mean equal to *reaction rate* $\times \Delta t$
  Sum the total changes in variables caused by stochastic each reaction over $(t,\ t + \Delta t)$ to obtain $\Delta_{stoch}\mathbf{v}$
  Set $\mathbf{v} = \mathbf{v} + \Delta_{det}\mathbf{v} + \Delta_{stoch}\mathbf{v}$
  for all entries v_j in $\mathbf{v}$ **do**
    if $v_j < 0$ **then**
      Set $v_j = 0$ to avoid negative molecule counts
    end if
  end for
  Set $t = t + \Delta t$
end while

are virtually unaffected by synthetic gene expression, which enables the analytical derivations in Supplementary Notes S3.5-S3.7 and S4.4.2. While no experimental evidence clearly in favor or against this simplification has been found to date, the mathematical reasoning behind our claim is outlined below.

Consider a cell expressing the set of synthetic genes $X = \{x_1,\ x_2,\ \dots x_L\}$ alongside its native genes. The resource competition denominator D, originally given by Equation (S29), in this case should also include synthetic genes, hence

$$D = 1 + \frac{1}{1 - \bar{\phi}_q} \sum_{j \in \{a,r\} \cup X} m_j / k_j \tag{S85}$$

Due to the finite-proteome cellular trade-off, its growth rate $\lambda(\epsilon, B)$ is equal to the total rate of protein translation by all mRNA-bound ribosomes (see Equation (S8)). The share of ribosomes that are bound to transcripts can in turn be calculated from D according to Equation (S19). Therefore, the cell's growth rate can be redefined in terms of the resource competition denominator and the overall abundance of ribosomes R.

$$\lambda(\epsilon, B) = \frac{\epsilon(t^c) \cdot B}{M} = \frac{\epsilon(t^c)}{M} \cdot R \cdot \left(1 - \frac{1}{D}\right) \tag{S86}$$

Let us now consider the ODE for the concentration of uncharged tRNAs (Equa-

tion (S73)) in steady state.

$$0 = \dot{t}^u = \psi(\overline{T}) \cdot \lambda - \nu(\overline{t}^u, \sigma) \cdot \overline{p}_a + \epsilon(\overline{t}^c) \cdot \overline{B} - \lambda(\epsilon, \overline{B}) \cdot t^u \tag{S87}$$

Substituting Equation (S86) into it, we get:

$$0 = \psi(\overline{T}) \cdot \left(\frac{\epsilon(\overline{t}^c)}{M} \cdot \overline{R} \cdot (1 - \frac{1}{\overline{\overline{D}}}) \right) - \nu(\overline{t}^u, \sigma) \cdot \overline{p}_a + \epsilon(\overline{t}^c) \cdot \overline{R} \cdot (1 - \frac{1}{\overline{\overline{D}}}) - t^u \cdot \left(\frac{\epsilon(\overline{t}^c)}{M} \cdot \overline{R} \cdot (1 - \frac{1}{\overline{\overline{D}}}) \right) \tag{S88}$$

A crucial feature of Equation (S88) is that all of its terms include a ribosomal or metabolic protein concentration. The steady-state mRNA concentrations of these genes can be found as:

$$\overline{m}_a = \frac{c_a \alpha_a \overline{\lambda}}{\beta_a + \overline{\lambda}} \tag{S89}$$

$$\overline{m}_r = \frac{F_r(\overline{T}) c_r \alpha_r \overline{\lambda}}{\beta_r + \overline{\lambda}} \tag{S90}$$

Let us now remember Equation (S77), which expresses the steady-state concentration of gene i's protein in terms of the cell's growth rate, RC denominator and gene i's parameters, mRNA concentration and apparent transcript-ribosome dissociation constant $k_i(\epsilon)$. Using it, we can substitute $\overline{p}_a$ and $\overline{R}$ in Equation (S88) as shown below.

$$\begin{aligned} 0 = \psi(\overline{T}) \cdot \left(\frac{\epsilon(\overline{t}^c)}{M} \cdot (1 - \frac{1}{\overline{\overline{D}}}) \cdot \frac{F_r(\overline{T}) c_r \alpha_r \overline{\lambda}}{k_r(\epsilon)(\beta_r + \overline{\lambda})} \cdot \frac{1}{\overline{\overline{D}}} \right) - \\ - \nu(\overline{t}^u, \sigma) \cdot \frac{c_a \alpha_a \overline{\lambda}}{k_a(\epsilon)(\beta_a + \overline{\lambda})} \cdot \frac{1}{\overline{\overline{D}}} + \\ + \epsilon(\overline{t}^c) \cdot (1 - \frac{1}{\overline{\overline{D}}}) \cdot \frac{F_r(\overline{T}) c_r \alpha_r \overline{\lambda}}{k_r(\epsilon)(\beta_r + \overline{\lambda})} \cdot \frac{1}{\overline{\overline{D}}} - t^u \cdot \left(\frac{\epsilon(\overline{t}^c)}{M} \cdot (1 - \frac{1}{\overline{\overline{D}}}) \cdot \frac{F_r(\overline{T}) c_r \alpha_r \overline{\lambda}}{k_r(\epsilon)(\beta_r + \overline{\lambda})} \cdot \frac{1}{\overline{\overline{D}}} \right) \end{aligned} \tag{S91}$$

Given that in our model $\beta_a = \beta_r$, Equation (S91) can be simplified to:

$$\begin{aligned} 0 = \psi(\overline{T}) \cdot \left(\frac{\epsilon(\overline{t}^c)}{M} \cdot (1 - \frac{1}{\overline{\overline{D}}}) \cdot \frac{F_r(\overline{T}) c_r \alpha_r}{k_r(\epsilon)} \right) - \nu(\overline{t}^u, \sigma) \cdot \frac{c_a \alpha_a}{k_a(\epsilon)} + \\ + \epsilon(\overline{t}^c) \cdot (1 - \frac{1}{\overline{\overline{D}}}) \cdot \frac{F_r(\overline{T}) c_r \alpha_r}{k_r(\epsilon)} - t^u \cdot \left(\frac{\epsilon(\overline{t}^c)}{M} \cdot (1 - \frac{1}{\overline{\overline{D}}}) \cdot \frac{F_r(\overline{T}) c_r \alpha_r}{k_r(\epsilon)} \right) \end{aligned} \tag{S92}$$

According to Equation (S85), the resource competition denominator $\overline{D}$ is the sum of all mRNA concentrations (adjusted for multiple ribosomes translating the same mRNA), which for the native genes are on the order of magnitude of 10^4 - 10^5 nM, scaled by the apparent mRNA-ribosome dissociation constants which have the order of magnitude of 10^0 - 10^1 nM. With ${}^{m_a}/_{k_a}$ and ${}^{m_r}/_{k_r}$ therefore being large, any additional terms for synthetic mRNAs being non-negative, and the scaling coefficient $\frac{1}{1-\overline{\phi}_q} = \frac{1}{0.41} > 1$ it is reasonable to suggest that $\overline{D} \gg 1$. This reduces $\frac{1}{\overline{D}}$ to 0 and transforms the steady-state ODE for the uncharged tRNA concentration into Equation (S93).

$$0 = \psi(\overline{T}) \cdot \left(\frac{\epsilon(\overline{t}^c)}{M} \cdot \frac{F_r(\overline{T}) c_r \alpha_r}{k_r(\epsilon)} \right) - \nu(\overline{t}^u, \sigma) \cdot \frac{c_a \alpha_a}{k_a(\epsilon)} + \epsilon(\overline{t}^c) \cdot \frac{F_r(\overline{T}) c_r \alpha_r}{k_r(\epsilon)} - t^u \cdot \left(\frac{\epsilon(\overline{t}^c)}{M} \cdot \frac{F_r(\overline{T}) c_r \alpha_r}{k_r(\epsilon)} \right) \tag{S93}$$

Following the same steps, the steady-state ODE for the aminoacyl-tRNA concentration t^c can be reduced to Equation (S94).

$$0 = \nu(\overline{t}^u, \sigma) \cdot \frac{c_a \alpha_a}{k_a(\epsilon)} - \epsilon(\overline{t}^c) \cdot \frac{F_r(\overline{T}) c_r \alpha_r}{k_r(\epsilon)} - t^u \cdot \left(\frac{\epsilon(\overline{t}^c)}{M} \cdot \frac{F_r(\overline{T}) c_r \alpha_r}{k_r(\epsilon)} \right) \tag{S94}$$

While complex in form, Equations (S93)-(S94) only have two unknowns – the steady-state tRNA concentrations $\overline{t}^u$ and $\overline{t}^c$ (the ppGpp concentration stand-in $\overline{T}$ is defined as $\frac{\overline{t}^c}{\overline{t}^u}$). Moreover, they do not contain any terms dependent on synthetic gene expression. Therefore, the steady-state $\overline{t}^u$ and $\overline{t}^c$ values, which comprise the solution to the system of these two equations, are the same regardless of which, if any, synthetic genes are present in the cell.

Since the translation elongation rate $\epsilon(t^c)$ is a function of the aminoacyl-tRNA concentration, whereas the ribosomal and tRNA gene transcription regulation function $F_r(T)$ depends on the ratio of the aminoacylated and uncharged tRNA levels, their steady-state values are likewise unaffected by the gene expression burden. In Supplementary Figure S3, we confirm these findings numerically by finding the steady-state values of ϵ and $F_r(T)$ for different nutrient qualities σ and heteorologous mRNA production rates of up to twice the highest possible combined rate of ribosomal and metabolic gene transcript synthesis. Indeed, for either of the two physiological variables, the changes in the numerically obtained steady-state values are on the level of 10^{-6}.

S3.5 Estimating steady-state protein mass fractions with heterologous expression

If we apply the simplifying assumption from the previous Supplementary Note S3.4 to (Model VI), it becomes possible to derive many useful analytical relations regarding the host cell state's interdependence with burden, as well as to analytically predict the performance of synthetic gene circuits in light of resource competition. These derivations are presented in Supplementary Notes S3.5-S3.7 and S4.4.2. To begin with, here we find resource- and burden-aware estimates of the steady-state abundances of synthetic proteins in the cell.

In Supplementary Note S3.4 we showed that the ribosomal gene transcription regulation function F_r and the translation elongation rate ϵ are almost independent of the heterologous gene expression burden and the parameters describing heterologous gene expression. Using the index ^{NB} to denote the "no burden" steady state values of variables in absence of heterologous gene expression, this assumption can be written as

$$\epsilon \approx \bar{\epsilon}^{NB}, \qquad F_r \approx \overline{F}_r^{NB} \qquad \forall\{a_{x_l}\}, \{b_{x_l}\}, \{c_{x_l}\}, \{F_{x_l}\} \tag{S95}$$

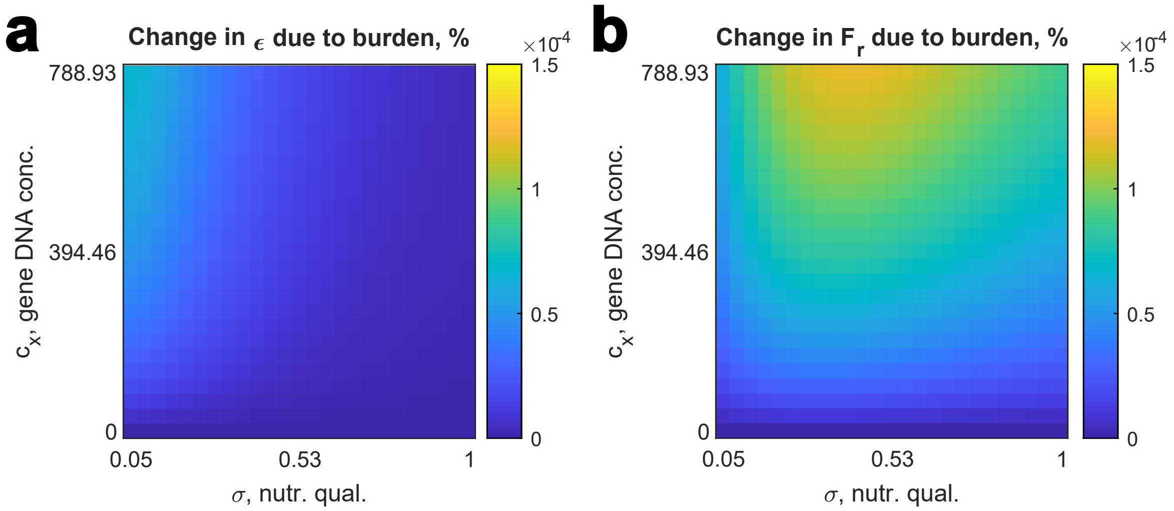

Supplementary Figure S3: **Magnitudes of changes in the steady-state values of cellular variables due to burden**. **a** Magnitudes of changes in the steady-state translation elongation rate $\bar{\epsilon}$ caused by expressing a heterologous gene for various heterologous DNA concentrations and culture medium's nutrient qualities. **b** Magnitudes of changes in the steady-state ribosome transcription regulation function $\overline{F}_r$ caused by expressing a heterologous gene for various heterologous DNA concentrations and culture medium's nutrient qualities. The heterologous gene's parameters are given in Supplementary Table S4. Source data are provided as a Source Data file.

where $\overline{\epsilon}^{NB}$ and $\overline{F}_r^{NB}$ are easily determined by numerically retrieving the steady state of the host cell expressing no synthetic genes (i.e., simulating (Model III) for a given value of σ). A corollary to that assumption is the notion that the mRNA-ribosome affinities are also burden-independent, as we show in Equation (S96).

$$k_i = k_i(\epsilon) = \frac{k_i^- + \epsilon/n_i}{k_i^+} \approx \frac{k_i^- + \overline{\epsilon}^{NB}/n_i}{k_i^+} = \overline{k}_i^{NB} \tag{S96}$$

In this case, what is the steady-state mRNA concentration for a given gene, be it native or heterologous? From Equations (S70) and (S75), it follows that

$$\overline{m}_i = \frac{\overline{F}_i c_i \alpha_i \lambda(\overline{\epsilon}^{NB}, \overline{B})}{\beta_i + \lambda(\overline{\epsilon}^{NB}, \overline{B})} \quad \forall i \in \{a, r\} \cup X \tag{S97}$$

where $\overline{F}_i$ is the steady-state value of gene i's regulatory function F_i. Provided that the mRNA decay rate is roughly the same for all synthetic genes and native gene classes – that is, $\beta_i \approx \beta_j \;\; \forall i, j \in \{a, r\} \cup X$ – the expression from Equation (S78) for the steady-state protein mass fraction can be rewritten to only include the gene transcription parameters and mRNA-ribosome affinities:

$$\overline{\phi}_i = \frac{\overline{m}_i/\overline{k}_i^{NB}}{\frac{1}{1-\overline{\phi}_q}\sum_{j\in\{a,r\}\cup X} \overline{m}_j/\overline{k}_j^{NB}} = \frac{\dfrac{\overline{F}_i c_i \alpha_i \lambda(\overline{\epsilon}^{NB}, \overline{B})}{(\beta + \lambda(\overline{\epsilon}^{NB}, \overline{B}))\cdot \overline{k}_i^{NB}}}{\dfrac{1}{1-\overline{\phi}_q}\displaystyle\sum_{j\in\{a,r\}\cup X} \dfrac{\overline{F}_j c_j \alpha_j \lambda(\overline{\epsilon}^{NB}, \overline{B})}{(\beta + \lambda(\overline{\epsilon}^{NB}, \overline{B}))\cdot \overline{k}_j^{NB}}} \Leftrightarrow$$

$$\Leftrightarrow \overline{\phi}_i = (1-\overline{\phi}_q)\cdot \frac{\overline{F}_i c_i \alpha_i/\overline{k}_i^{NB}}{\displaystyle\sum_{x_l\in X} \overline{F}_{x_l} c_{x_l} \alpha_{x_l}/\overline{k}_{x_l}^{NB} + \sum_{j\in\{a,r\}} \overline{F}_j c_j \alpha_j/\overline{k}_j^{NB}} \tag{S98}$$

Importantly, under the assumption of unchanging translation elongation rate and ppGpp signal regulating ribosomal gene transcription, Equation (S98)'s terms for both ribosomal and metabolic genes, which together constitute the set $\{a, r\}$, are constant and independent of the identity and parameters of the heterologous genes being expressed. Let us then define ξ, the "translational burden" or "translational demand" of heterologous gene expression – that is, the extent of competition for ribosomes collectively imposed by

all synthetic genes. This can be found by calculating the product of the copy number, promoter strength and mRNA-ribosome dissociation constant for every heterologous gene and adding these quantities together, as shown in Equation (S99).

$$\xi = \sum_{x_l \in X} \frac{\overline{F}_{x_l} c_{x_l} \alpha_{x_l}}{\overline{k}^{NB}_{x_l}} \tag{S99}$$

Consequently, the total mass fraction of all heterologous genes $\overline{\phi}_X$ follows a relationship akin to the Hill activation function:

$$\overline{\phi}_X(\xi) = \sum_{x_l \in X} \overline{\phi}_{x_l} = \frac{\xi}{\xi + (\sum_{j \in \{a,r\}} \overline{F}^{NB}_j c_j \alpha_j / \overline{k}^{NB}_j)} \tag{S100}$$

The mass fraction of an individual gene is therefore given by:

$$\overline{\phi}_{x_l}(\xi) = \overline{\phi}_X(\xi) \cdot \frac{\overline{F}_{x_l} c_{x_l} \alpha_{x_l} / \overline{k}^{NB}_{x_l}}{\sum_{j \in X} \overline{F}_j c_j \alpha_j / \overline{k}^{NB}_j} = \overline{\phi}_X(\xi) \cdot \frac{\overline{m}_{x_l} / \overline{k}_{x_l}}{\sum_{j \in X} \overline{m}_j / \overline{k}_j} \qquad \forall x_l \in X \tag{S101}$$

As for the native genes a and r, substitution into Equation (S98) shows that their mass fractions, conversely, obey a Hill repressor function-like law:

$$\overline{\phi}_i(\xi) = (1 - \overline{\phi}_q) \cdot \frac{\overline{F}^{NB}_i c_i \alpha_i / \overline{k}^{NB}_i}{\xi + \sum_{j \in \{a,r\}} \overline{F}_j c_j \alpha_j / \overline{k}^{NB}_j} \quad \forall i \in \{a, r\} \tag{S102}$$

S3.6 Estimating steady-state growth rate with heterologous gene expression

From the previous section, we have an intuition how the steady-state protein mass fractions are affected by the translational burden of expressing heterologous genes. We now use this knowledge to find how burden influences cell growth. Let us recall the definition of growth rate λ from Supplementary Table S2. In steady state, it yields:

$$\overline{\lambda} = \lambda(\overline{\epsilon}^{NB}, \overline{B}) = \frac{\overline{\epsilon}^{NB} \cdot \overline{B}}{M}$$

Let us assume that the share of free ribosomes is negligible – indeed, even without heterologous mRNA transcription, at high nutrient qualities D is very high (on the order of 10^4), so the share of ribosomes not engaged in translation, $\frac{1}{1+D}$, is insignificant. Then, we

have $B \approx R$, which transforms the expression for the steady-state growth rate into

$$\overline{\lambda} \approx \frac{\overline{\epsilon}^{NB} \cdot \overline{R}}{M} \Leftrightarrow$$

$$\Leftrightarrow \overline{\lambda}(\xi) = \frac{\overline{\epsilon}^{NB}}{n_r} \cdot \overline{\phi}_r(\xi) = \frac{\overline{\epsilon}^{NB}(1-\overline{\phi}_q)}{M} \cdot \frac{\overline{F}_r^{NB} c_r \alpha_r / \overline{k}_r^{NB}}{\xi + \sum_{j \in \{a,r\}} \overline{F}_j^{NB} c_j \alpha_j / \overline{k}_j^{NB}} \tag{S103}$$

This is similar in form to the Hill relationship between the heterologous gene expression burden and the steady-state growth rate observed by McBride et al. [1]. Meanwhile, the ratio between the growth rates with and without heterologous gene expression burden can be found as shown in Equation (S104). This matches the linear-like relationship between the growth rate and heterologous protein mass fraction observed by Scott et al. [10].

$$\frac{\overline{\lambda}(\xi)}{\overline{\lambda}^{NB}} \approx \left(\frac{\overline{\epsilon}^{NB}(1-\overline{\phi}_q)}{M} \cdot \frac{\overline{F}_r^{NB} c_r \alpha_r / \overline{k}_r^{NB}}{\xi + \sum_{j \in \{a,r\}} \overline{F}_j^{NB} c_j \alpha_j / \overline{k}_j^{NB}} \right) \div \left(\frac{\overline{\epsilon}^{NB}(1-\overline{\phi}_q)}{M} \cdot \frac{\overline{F}_r^{NB} c_r \alpha_r / \overline{k}_r^{NB}}{\sum_{j \in \{a,r\}} \overline{F}_j^{NB} c_j \alpha_j / \overline{k}_j^{NB}} \right) \Leftrightarrow$$

$$\Leftrightarrow \frac{\overline{\lambda}(\xi)}{\overline{\lambda}^{NB}} \approx \frac{\sum_{j \in \{a,r\}} \overline{F}_j^{NB} c_j \alpha_j / \overline{k}_j^{NB}}{\xi + \sum_{j \in \{a,r\}} \overline{F}_j^{NB} c_j \alpha_j / \overline{k}_j^{NB}} = 1 - \frac{\xi}{\xi + \sum_{j \in \{a,r\}} \overline{F}_j^{NB} c_j \alpha_j / \overline{k}_j^{NB}} \Leftrightarrow$$

$$\Leftrightarrow \frac{\overline{\lambda}(\xi)}{\overline{\lambda}^{NB}} \approx 1 - \frac{\overline{\phi}_X(\xi)}{1-\overline{\phi}_q} \tag{S104}$$

S3.7 Maximizing heterologous protein production

A prominent application of synthetic biology is bioproduction of valuable proteins [21]. In order to ensure the process's cost-efficiency, it is thus desirable to maximise the yield of the protein produced by a population of cells in a bioreactor. A key hindrance to achieving this, however, is the need to balance high protein synthesis rates with small gene expression burden. Indeed, while a single cell can be made to produce a lot of

protein, severe impairment of growth by high heterologous gene expression would mean that the cell population expands very slowly, so the overall rate of protein production would in fact be low [22].

The relations derived in Supplementary Note S3.5 and S3.6 can be leveraged to address this issue. Let us define a simple model of a homogeneous population of *E. coli* cells, dying at a constant rate δ [2], where N denotes the overall number of cells and λ is the cell growth rate as predicted by our model (Equation (S105)).

$$\dot{N} = \lambda N - \delta N \tag{S105}$$

If the bacteria express a single gene *poi* encoding the intracellular protein of interest and are in steady state, how would we approach maximizing the protein yield? Protein production per cell is commonly characterized as the product of the amount of protein in one cell and the rate at which the population expands [23]. Under our model, this is equal to:

$$\mu = M \cdot \overline{\phi}_{poi} \cdot (\overline{\lambda} - \delta) \tag{S106}$$

Since the steady-state growth rate can be estimated with the help of Equation (S104), we can make a substitution to obtain:

$$\begin{aligned} \mu \approx M \cdot \overline{\phi}_{poi} \cdot \left(\overline{\lambda}^{NB} \left(1 - \frac{\overline{\phi}_{poi}}{1 - \overline{\phi}_q} \right) - \delta \right) \Leftrightarrow \\ \Leftrightarrow \mu \approx - \frac{\overline{\lambda}^{NB}}{1 - \overline{\phi}_q} M \overline{\phi}_{poi}^2 + M(\overline{\lambda}^{NB} - \delta)\overline{\phi}_{poi} \end{aligned} \tag{S107}$$

This is a quadratic expression, so the value of $\overline{\phi}_{poi}$ that maximizes it can be easily calculated as:

$$\overline{\phi}_{poi}^{max} = \frac{1}{2} \left(1 - \overline{\phi}_q \right) \left(1 - \frac{\delta}{\overline{\lambda}^{NB}} \right) \tag{S108}$$

Knowing this value, we can use Equation (S100) to find the translational burden ξ_{max} corresponding to the optimal heterologous protein mass fraction. This allows to ensure maximum protein production by imposing a condition on the synthetic gene's mRNA-ribosome dissociation constant, gene copy number and promoter strength, which

we provide below.

$$
\begin{aligned}
&\overline{\phi}_{poi}(\xi_{max}) = \overline{\phi}_{poi}^{max} \Leftrightarrow \\
&\Leftrightarrow \frac{c_{poi}\alpha_{poi}}{\overline{k}_{poi}^{NB}} = \xi_{max} = \frac{1-\delta/\overline{\lambda}^{NB}}{1+\delta/\overline{\lambda}^{NB}} \cdot \sum_{j\in\{a,r\}} \frac{\overline{F}_j c_j \alpha_j}{\overline{k}_j^{NB}}
\end{aligned}
\tag{S109}
$$

S4 Biocircuit modeling

In this section, we define the ODEs for the synthetic gene circuits considered in the article. We also provide derivations of the analytical relations that can guide biocircuit design and show the results of simulations that were conducted in addition to those described in the main text.

S4.1 Feasible synthetic gene parameter ranges

In order to simulate the circuit ODEs, the parameters found in them must be assigned with realistic values. For the sake of simplicity, generic values (equal to those for the native metabolic genes) were chosen for all synthetic gene lengths $\{n_{x_l}\}$ and mRNA degradation rates $\{\beta_{x_l}\}$, as well as mRNA-ribosome binding and dissociation rates $\{k^+_{x_l}\}$ and $\{k^-_{x_l}\}$. All synthetic genes were also assumed to be hosted by high-copy number plasmids, hence us setting the gene concentrations to be $\{c_{x_l} = 100\ nM\}$ when gene copy numbers were not varied.

Numerous constitutive [24], inducible [25, 26], and repressible [27] synthetic promoters of different strengths have been defined and characterized over the past years. To define the feasible range of strengths for a given type of promoter, we therefore considered the weakest and the strongest promoter in a corresponding published library. Promoter strengths for these collections were given in relative promoter units (RPU) with respect to the J23101 promoter. While the exact conversion between RPU and the absolute rate of mRNA synthesis is dependent on measurement conditions, a rough order-of-magnitude estimate makes 1 RPU equivalent to 0.03 mRNA molecules being transcribed per second [24]. The promoter strengths in RPU were thus converted into our dimensionless α values by estimating the absolute mRNA synthesis rates, then dividing them by the cell growth rate at the time of measurement (since in our model the rate of transcription is proportional that of cell growth), and finally scaling them by ${}^{n_{x_l}}/_{25} = {}^{300}/_{25}$ to account for the translation of a single transcript by multiple ribosomes as outlined in Supplementary Note S2.3. In line with our choice of gene concentrations, when promoter strengths were separately measured for plasmids with different copy numbers [27], we used the data for the highest-copy number plasmids. The calculated feasible promoter strength ranges, along with the realistic ranges of values for other parameters characterizing synthetic gene

transcription regulation, are displayed in Supplementary Table S5.

An exception to the considerations in this section is the T7 RNA polymerase (*T7 RNAP*) gene *t7*, whose length $n_{t7} = 883\ aa$ is much larger than the generic value of $300\ aa$, and mRNA degradation rate is known to be different from those of *E. coli*'s native proteins [20]. As for the plasmid copy number, the toxicity of T7 RNAP made us choose a low-copy number plasmid ($c_{t7} = 5\ nM$) as a more realistic vector for it.

Supplementary Table S5: **Feasible ranges for the synthetic gene circuit simulation parameters.**

Param.	Description	Min.	Max.	Units	Source
α_{x_l}	Constitutive promoter strength	40	2,500	nM	[24]
α_{x_l}	Inducible promoter strength	35.8	6,700	nM	[25]
α_{x_l}	Repressible promoter strength	320	4,750	nM	[27]
$K_{dna_{x_l}}$	Half-saturation constant for protein-inducer binding	10^{-2}	10^{4}	nM	[7]
$K_{ind_{x_l}}$	Half-saturation constant for transcription factor-inducer binding	10^{-2}	10^{4}	nM	[7]
$F_{x_l,0}$	Gene transcription regulation function value in absence of inducer	≈ 0	0.2	None	[26]
η_{x_l}	Cooperativity coefficient for the transcription factor-promoter DNA binding	1	5	None	[26]

S4.2 Two bistable switches exhibiting "winner-takes-all" behavior

S4.2.1 Description

To demonstrate that our model captures known resource competition phenomena, we considered the case of two synthetic self-activating genes being present in the same cell. Here, "self-activation" means that the protein encoded by a gene can bind a corresponding inducer molecule to form a complex that acts as a transcription activation (TA) factor for the same gene. On its own, such a gene can act as a bistable switch due to having two different stable steady states, one with high gene expression and the other with low expression. Meanwhile, two switches in the same cell may exhibit "winner-takes-all" behavior – that is, if one gene reaches its high-expression equilibrium faster than the other,

the increased resource competition from it can prevent the second gene from becoming highly expressed [28].

For simplicity, we characterized the two synthetic self-activating genes s_1 and s_2 by the same parameter values, displayed in Supplementary Table S6. The ODEs describing their expression mirrored the generic Equations (S75) and (S76) for heterologous mRNA and protein concentrations, hence:

$$\dot{m_{s_1}} = F_{s_1}(f_{s_1}, p_{s_1}) \cdot c_{s_1}\alpha_{s_1} - (\beta_{s_1} + \lambda(\epsilon, B))m_{s_1} \tag{S110}$$

$$\dot{p_{s_1}} = \frac{\epsilon(t^c)}{n_{s_1}} \cdot \frac{m_{s_1}/k_{s_1}}{1 + \frac{1}{1-\bar{\phi}_q}\sum_{j\in\{a,r\}\cup X} m_j/k_j} R - \lambda(\epsilon, B) \cdot p_{s_1} \tag{S111}$$

$$\dot{m_{s_2}} = F_{s_2}(f_{s_2}, p_{s_2}) \cdot c_{s_2}\alpha_{s_2} - (\beta_{s_2} + \lambda(\epsilon, B))m_{s_2} \tag{S112}$$

$$\dot{p_{s_2}} = \frac{\epsilon(t^c)}{n_{s_2}} \cdot \frac{m_{s_2}/k_{s_2}}{1 + \frac{1}{1-\bar{\phi}_q}\sum_{j\in\{a,r\}\cup X} m_j/k_j} R - \lambda(\epsilon, B) \cdot p_{s_2} \tag{S113}$$

where the transcription regulation function for gene s_i is defined as:

$$F_{s_i}(f_i, p_{s_i}) = \frac{F_{s_i,0} \cdot K_{dna_i}^{\eta_i} + \left(\frac{f_i}{K_{ind_i} + f_i} \cdot p_{s_i}\right)^{\eta_i}}{K_{dna_i}^{\eta_i} + \left(\frac{f_i}{K_{ind_i} + f_i} \cdot p_{s_i}\right)^{\eta_i}} \tag{S114}$$

Here, f_i is the inducer concentration. To simulate the activation of a switch at time $t_{induction}$, we made it follow a step function of time t (Equation (S115)). In order to investigate different possible behaviors of the system, different combinations of the two genes' added inducer concentrations, f_1 and f_2, were considered as outlined in the main text.

$$f_i(t) = \begin{cases} 0, \text{ if } t < t_{induction} \\ f_i, \text{ otherwise} \end{cases} \tag{S115}$$

S4.2.2 Additional simulations

For a system of two self-activating bistable switch genes in the same host cell, additional simulations were performed besides those discussed in the main text's Results section and displayed in the main text's Figure 4b. The details of these numerical experiments are outlined in this section.

Supplementary Table S6: **Parameters of the synthetic self-activating genes.** Parameter values were chosen in line with the feasible ranges from Supplementary Table S5.

Parameter	Description	Value	Units
c_{s_i}	Gene copy number	100	nM
α_{s_i}	Promoter strength	3,000	None
β_{s_i}	mRNA degradation rate	6	h^{-1}
$k^+_{s_i}$	mRNA-ribosome binding rate	60	$\frac{1}{nM \cdot h}$
$k^-_{s_i}$	mRNA-ribosome dissociation rate	60	h^{-1}
n_{s_i}	Number of amino acids in protein p_i	300	aa/nM
	**Gene transcription regulation function**		
$F_{s_i,0}$	Baseline function value in absence of inducer	0.05	None
K_{ind_i}	Half-saturation constant for protein-inducer binding	$1,000$	nM
K_{dna_i}	Half-saturation constant for the TA factor-promoter DNA binding	$5,000$	nM
η_i	Cooperativity coefficient for the TA factor-promoter DNA binding	2	None

In the main text, we discussed the winner-takes-all phenomenon exhibited by the described system, where the switch that reaches a high-expression equilibrium first (the "winner") prevents the other switch from becoming activated due to resource couplings. In Figure 4b, we demonstrated this by varying the concentration of the first self-activating gene's inducer, f_1. Since the gene with a higher inducer concentration becomes activated first, setting f_1 lower than that the second gene's inducer concentration f_2 led to the second switch "winning". Conversely, for $f_1 > f_2$ the first switch gene prevailed while the expression of the second remained low [28].

To demonstrate that tuning the inducer's concentration indeed allows to manipulate the timescale of the corresponding switch's activation, in Supplementary Figure S4a we plotted the evolution of a switch protein's concentration over time upon the addition of different concentrations of the inducer (f_1) to the medium. Indeed, it can be seen that higher f_1 values cause the system to converge to its high-expression equilibrium faster. Importantly, if resource competition from another synthetic gene prevents activation from ever occurring, observing its speed would be impossible. Supplementary Figure S4a therefore considers the case of a single bistable switch being present in the cell, whereas the concentration of the second switch gene's DNA is set to zero.

According to Supplementary Figure S4a, increasing the inducer's concentration not only speeds up a switch's activation, but also shifts the position of the high-expression

equilibrium towards greater protein concentrations. We therefore aimed to show that the winner-takes-all phenomenon is primarily enabled by the disparity in the switches' activation timescales rather than unequal protein expression levels in the activated state. To this end, one can activate the two switches in different order – but without altering the inducer levels – to make the system converge to qualitatively different steady states [28]. We reproduce this observation in Supplementary Figure S4b–d: while inducing both switch genes simultaneously leads to co-activation (Supplementary Figure S4c), activating one switch ahead of the other makes it converge to the high-expression equilibrium as the other switch remains unactivated due to the winner-takes-all phenomenon (Supplementary Figure S4b and d).

At the same time, the extent of switch protein expression must still have a significant effect on winner-takes-all behavior. Indeed, since the phenomenon in question is enabled by gene expression burden, resource competition exerted by the switches must be great enough to significantly affect gene expression. To investigate the relationship between winner-takes-all behavior and the burden caused by the switch genes, we repeated the simulation outlined above but scaled the genes' promoter strengths by a factor of $1/v$, since a weaker promoter means that fewer mRNA molecules are transcribed, so fewer transcripts compete for ribosomes. As decreasing transcription rates also reduces the overall amount of protein produced, the half-saturation constants for DNA-transcription factor binding were scaled, too – otherwise, p_{s_i} could be too low compared to K_{dna_i} for the switch to exhibit bistability. All other parameters remained as given in Supplementary Table S6. Supplementary Figure S4 shows that as v increases, the effect of higher inducer 1 concentrations on the second gene's expression becomes less significant. Accordingly, when $v = 5$ or $v = 10$, no system equilibria for $4\ nM \leq f_1 \leq 8000\ nM$ are associated with low p_{s_2} values. Hence, even a much faster-activated first switch cannot sequester enough translational resources to prevent the second switch from becoming activated. Numerical simulations conducted with the help of our model can therefore not only capture the well-known winner-takes-all resource competition phenomenon, but also allow to determine if it will be exhibited for a given combination of design parameters. However, it is important to note that *in vivo* a switch circuit is affected not only by resource couplings but also by other factors, such as the stochasticity of gene expression, which can also significantly alter its behavior [29].

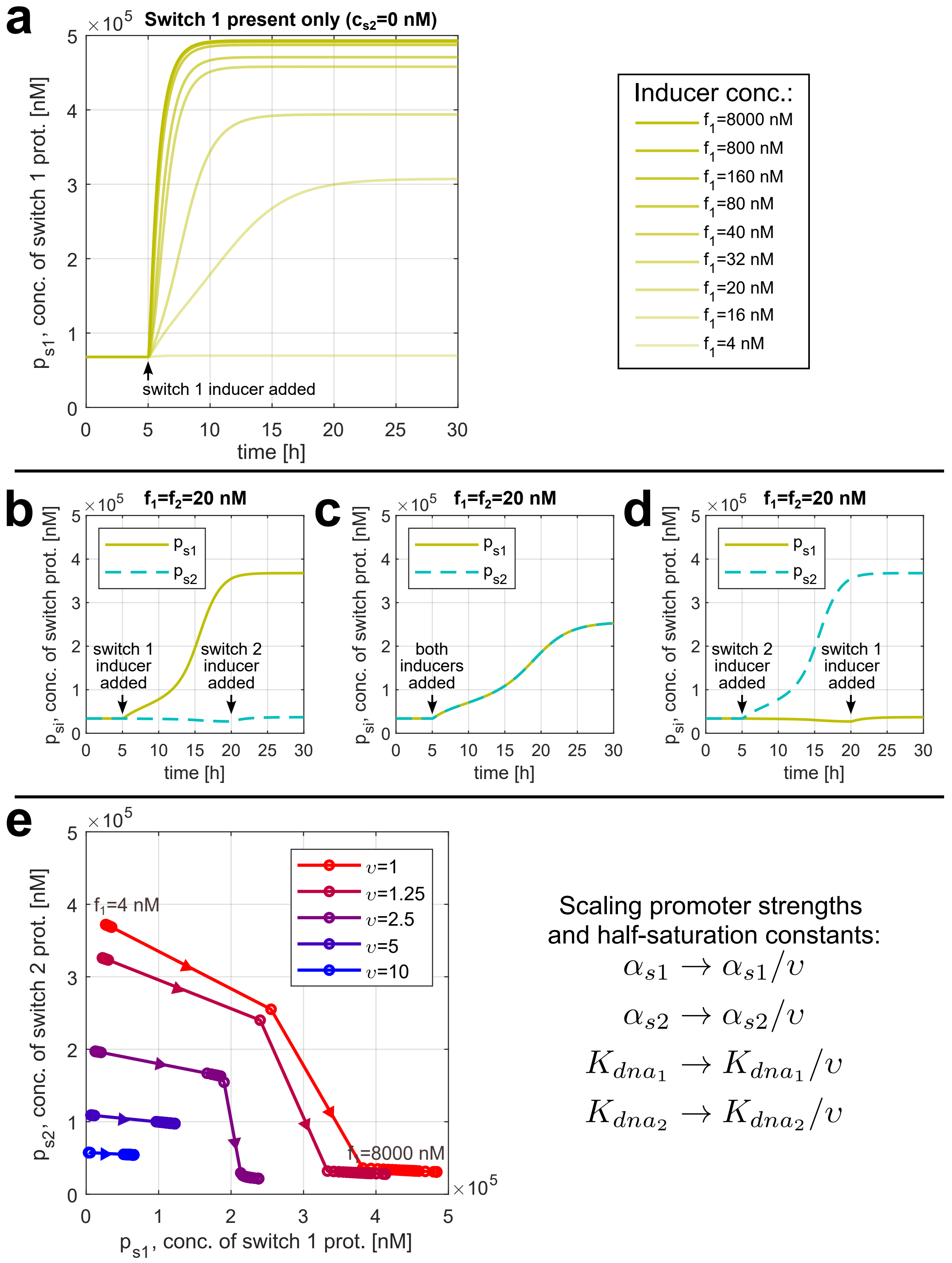
a
Switch 1 present only (cs2=0 nM)
×10^5
ps1, conc. of switch 1 prot. [nM]
time [h]
switch 1 inducer added
Inducer conc.:
f1=8000 nM
f1=800 nM
f1=160 nM
f1=80 nM
f1=40 nM
f1=32 nM
f1=20 nM
f1=16 nM
f1=4 nM
b
f1=f2=20 nM
psi, conc. of switch prot. [nM]
ps1
ps2
switch 1 inducer added
switch 2 inducer added
time [h]
c
f1=f2=20 nM
both inducers added
d
f1=f2=20 nM
switch 2 inducer added
switch 1 inducer added
e
ps2, conc. of switch 2 prot. [nM]
ps1, conc. of switch 1 prot. [nM]
f1=4 nM
f1=8000 nM
υ=1
υ=1.25
υ=2.5
υ=5
υ=10
Scaling promoter strengths
and half-saturation constants:
$\alpha_{s1} \to \alpha_{s1}/\upsilon$
$\alpha_{s2} \to \alpha_{s2}/\upsilon$
$K_{dna_1} \to K_{dna_1}/\upsilon$
$K_{dna_2} \to K_{dna_2}/\upsilon$

Supplementary Figure S4: **Additional simulations for the winner-takes-all phenomenon.** **a** Simulations of a single bistable switch in the host cell activated by different concentrations of the corresponding inducer molecule in the medium. **c–d** Simulation of a system of two bistable switches hosted by the same cell and activated in different order. In all cases, the concentrations of both inducers used to activate the switches were taken to be identical, i.e., $f_1 = f_2 = 20\ nM$. **e** Phase plane diagram for the system of two self-activating bistable switch genes with the promoter strengths and DNA-transcription factor binding half-saturation constants scaled by $1/\upsilon$ for different values of υ. Bold circles joined by lines show the system's steady states in presence of 20 nM of inducer 2 and concentrations of inducer 1 ranging between 4 nM and 8000 nM. The arrows on the lines point in the direction of increasing inducer 1's concentration, f_1. Unless stated otherwise, all circuit parameters were sourced from Supplementary Table S9. Source data are provided as a Source Data file.

S4.3 A bistable non-cooperative self-activator

S4.3.1 Description

To show how our model captures the interplay between synthetic gene circuit performance and the host cell's growth, we turned to the example of a non-cooperative self-activator (see the main text, Figure 4c). While the existence of two stable equilibria for a self-activating gene is conditional on cooperativity, experiments show that the slowdown of cell growth due to synthetic gene expression can introduce an additional feedback loop and lift this requirement [30]. Recreating this phenomenon, we therefore model the expression of a heterologous *T7 RNAP* gene by the *E. coli* cell.

The ODE for the corresponding mRNA's concentration m_{t7} is defined in the vein of Equation (S75) for generic heterologous transcripts with a single important distinction that the rate of mRNA synthesis is not proportional to the cell's growth rate (hence the units of the promoter strength α_{t7} being h^{-1}). Indeed, a likely source of this proportionality for the cell's native mRNA are changes in the availability of the σ^{70} factor [31], which enables the functioning of the bacterial RNAPs but is not relevant for the T7 phage RNAP [32]. This yields:

$$\dot{m}_{t7} = F_{x_l} \cdot c_{x_l} \alpha_{x_l} - (\beta_{x_l} + \lambda(\epsilon, B)) m_{x_l} \tag{S116}$$

The gene transcription regulation function for the gene captures the effect of T7 RNAP's abundance on the rate of mRNA synthesis with a Michaelis-Menten expression (Equation (S117)) [30], which is non-competitive because no other genes are transcribed

by the T7 RNAP.

$$F_{t7}(f_i, p_{t7}) = \frac{F_{t7,0} \cdot K_{t7} + p_{t7}}{K_{t7} + p_{t7}} \tag{S117}$$

Since the T7 RNAP decays in the cell at a rate comparable to that of cell growth, we modify the model to account for protein degradation as outlined in Supplementary Note S3.2. The ODE for the T7 RNAP protein concentration is therefore given by Equation (S118).

$$\dot{p_{t7}} = \frac{\epsilon(t^c)}{n_{t7}} \cdot \frac{m_{t7}/k_{t7}}{1 + \frac{\sum_{j\in\{a,r\}\cup X} m_j/k_j - \overline{\phi}_q \frac{\delta_{t7} n_{t7} p_{t7}}{M}}{1 - \overline{\phi}_q \left(1 - \delta_{t7} n_{t7} p_{t7}/M\right)}} \cdot R - \lambda p_{t7} - \delta_{t7} p_{t7} \tag{S118}$$

A key component of the non-cooperative self-activator's emergent bistability is its toxicity to the host cell [20]. While part of T7 RNAP-based gene expression systems' impact on the cell's viability can be attributed to their demand for ribosomes and tRNAs, which our cell model captures explicitly, they also interfere with the host's amino acid metabolism [33]. We account for the latter effect using a Hill relation, redefining the aminoacyl-tRNA synthesis rate as

$$\nu(t^u, \sigma) = \nu_{max} \cdot \sigma \cdot \frac{t^u}{t^u + K_\nu} \cdot \frac{1}{1 + \gamma_{tox} p_{t7}} \tag{S119}$$

where γ_{tox} is the inverse of the Hill constant quantifying the toxicity of the T7 RNAP for the cell. The values of this and other parameters characterizing this circuit are given in Supplementary Table S7.

S4.3.2 Additional simulations

In order to study whether the system's bistability depends on the toxicity of T7 RNAP, we started by setting γ_{tox} to different values and simulating the circuit's behavior for a wide range of initial synthetic protein concentrations. As it can be seen in Supplementary Figure S5a–b, when T7 RNAP is non-toxic or weakly toxic, all initial conditions – even $p_{t7} = 10^4\ nM$, which is four times greater than the high-expression fixed point in the main text's Figure 4d – converge to a low-expression equilibrium. When γ_{tox} is considerably greater than the value of 0.03 nM^{-1} from Supplementary Table S7, all trajectories, conversely, converge to a high-expression equilibrium (Supplementary Figure S5c). This

Supplementary Table S7: **Parameters of the *T7 RNAP* gene.**

Parameter	Description	Value	Units
c_{t7}	Gene copy number	5*	nM
α_{t7}	Promoter strength	170 [20]	h^{-1}
β_{t7}	mRNA degradation rate	16.64 [20]	h^{-1}
k^+_{t7}	mRNA-ribosome binding rate	60 [2]	$\frac{1}{nM \cdot h}$
k^-_{t7}	mRNA-ribosome dissociation rate	60 [2]	h^{-1}
n_{t7}	Number of amino acids in protein p_i	883 [20]	aa/nM
δ_{t7}	T7 RNAP protein degradation rate	0.18 [20]	h^{-1}
γ_{tox}	Toxicity of T7 RNAP	0.03 [20]	nM^{-1}
	**Gene transcription regulation function**		
$F_{t7,0}$	Baseline function value in absence of inducer	0.0016 [20]	None
K_{t7}	Dissociation constant between the T7 RNAP and the T7 promoter	550 [20]	nM

* Gene borne by a low-copy number plasmid.

hints that the non-cooperative self-activator is only bistable for a certain range of T7 RNAP toxicity values.

In order to obtain a more comprehensive bifurcation analysis and ensure that our results in Supplementary Figure S5a–c were not merely caused by shifts in the boundary between the two stable equilibria's basins of attraction, we turned to control-based continuation (CBC). Recently established as a means for studying biological systems [34], it allows to trace a system's bifurcation curve for a given parameter. To this end, the bifurcation parameter (in our case, γ_{tox}) is treated as a control input, which enacts feedback to drive a state variable (in our case, p_{t7}) to a desired reference value. Once the system converges to a steady state – possibly with a non-zero error between the state and the reference if solely proportional feedback is applied – its position on the bifurcation diagram is recorded and the next reference value is set. Due to feedback's stabilizing effect, the obtained sequence of recorded steady states yields the system's both stable and unstable equilibria, therefore allowing to reconstruct its bifurcation curve.

Our CBC algorithm, summarized in Algorithm S2, was implemented as a Matlab script which can be found with the rest of our code at `https://github.com/KSechkar/rc_e_coli` [35]. Notably, our program is not specific to the T7 RNAP circuit and can be easily modified to numerically probe an arbitrary circuit's bifurcations with respect to a given parameter, which can be particularly useful in studying burden-related phenomena. The bifurcation diagram obtained by running this code for our case is displayed

in Supplementary Figure S5d, whereas the reference values, the CBC trajectory and the feedback gains used are given by Supplementary Figure S5e–g.

The bifurcation curve in Supplementary Figure S5d is consistent with the system being bistable only for a particular range of parameter values [34]. Starting with a single low-expression equilibrium when γ_{tox} is zero or very small (as we see in Supplementary Figure S5a–b), it then undergoes a saddle-node bifurcation at around 0.007 nM^{-1}, so three equilibria arise. The ones at the highest and lowest p_{t7} values are stable, whereas the middle equilibrium is unstable and serves as a boundary between the other two fixed points' basins of attraction (but can be retrieved by CBC due to stabilizing feedback). This region – which contains $\gamma_{tox} = 0.03$ that gave rise to the main text's Figure 4d–e – is where the phenomenon of burden-induced emergent bistability is observed. As γ_{tox} is increased further, another bifurcation occurs at $\gamma_{tox} \approx 0.109$ nM^{-1}, rendering the system monostable once more, but now with the equilibrium at high p_{t7} values, like we see in Supplementary Figure S5c. These observations are consistent both with the results obtained by Tan et al. [30] who identified the phenomenon in question, and with prior cell modeling studies that aimed to capture the behavior of a self-transcribing T7 RNAP in the host cell [20].

Algorithm S2 CBC algorithm used with our model. Here, $P_{t7} = \{p_{t7}^{ref,1},\ p_{t7}^{ref,2},\ \dots\ , p_{t7}^{ref,J}\}$ is the list of reference values tracked by CBC; K_p^j is the feedback gain used for the reference $p_{t7}^{ref,j}$; t is the simulation time in hours; $\mathbf{v}$ is the vector capturing the state of the entire system like in Algorithm S1. The results in Supplementary Figure S5 were obtained with the sampling step $\Delta t = 0.05\ h$, initial simulation time $t_{init} = 960\ h$ and initial toxicity $\gamma_{tox} = 0.13\ nM^{-1}$. For a given reference, the CBC system was assumed to reach equilibrium after having been simulated for a fixed time [34] $t_{eq} = 240\ h$ $(4,800$ sampling steps).

Get the system's steady state $\mathbf{v}_{init}$ for $\gamma_{tox} = \gamma_{tox}^{init}$ by simulating it for t_{init} hours
Set $\mathbf{v} = \mathbf{v}_{init}$, $\gamma_{tox} = \gamma_{tox}^{init}$, $t = 0\ h$, $j = 1$
while $j \leq J$ **do**
  while $t < j \times t_{eq}$ **do**
    Get p_{t7} from $\mathbf{v}$
    Set $\gamma_{tox} = K_p^j(p_{t7}^{ref,j} - p_{t7})$
    Update $\mathbf{v}$ by simulating the system for Δt hours, starting at the old $\mathbf{v}$
    Set $t = t + \Delta t$
  end while
  Get p_{t7} from $\mathbf{v}$
  Record $E_j = (p_{t7}, \gamma_{tox})$ as the system's equilibrium for reference $p_{t7}^{ref,j}$
end while

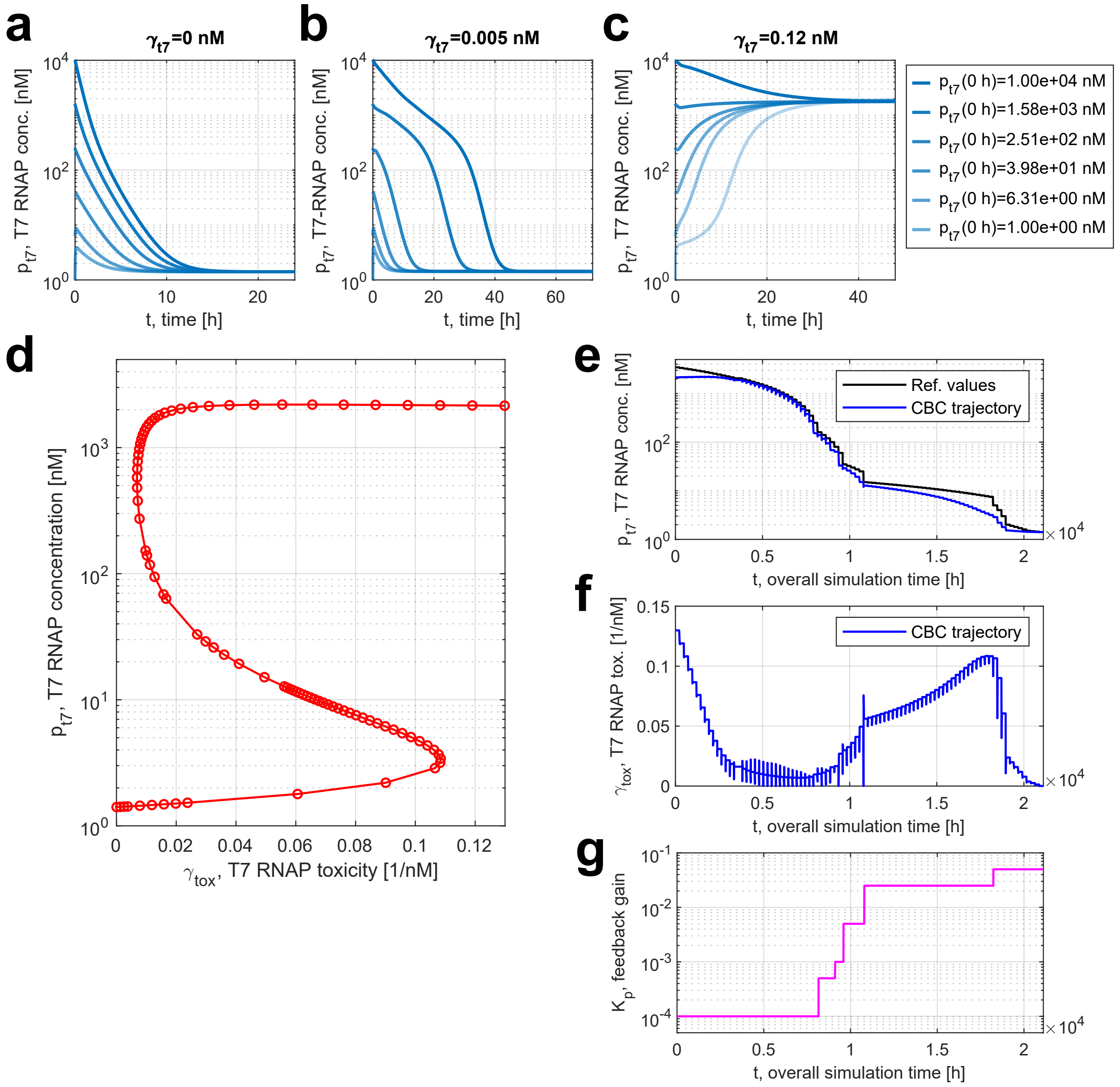

Supplementary Figure S5: **Dependence of the non-cooperative self-activator's equilibria on T7 RNAP's toxicity.** **a–c** Simulations of the system's behavior for different γ_{tox} values starting at $m_{t7} = 5\ nM$ and six different initial concentrations of p_{t7}, logarithmically spaced between $1\ nM$ and $10^4\ nM$. **d** The non-cooperative self-activator's bifurcation curve for γ_{tox} values. **e** CBC time evolution of p_{t7} and the corresponding control reference signal. **f** CBC time evolution of γ_{tox}. **g** Proportional feedback gains used over the course of CBC simulations – due to the lack of general and systematic methods for CBC controller design [34], this value was determined by trial and error for each reference so as to yield stable. All simulation parameters besides γ_{tox} were taken from Supplementary Table S7. Source data are provided as a Source Data file.

S4.4 An integral feedback controller for mitigating burden

S4.4.1 Description

Here, we consider the antithetic integral feedback (AIF) controller that maintains a constant level of competition for ribosomes in the cell and thus a constant growth rate. This circuit's working principle is outlined in the main text's Results section.

Briefly, it consists of four genes: the "sensor" *sens*, the "actuator" *act*, the "annihilator" *anti* and the "amplifier" *amp*. The actuator is a constitutive gene, whose mRNA m_{act} can be translated to produce the regulatory protein p_{act} that activates the expression of the amplifier gene's mRNA m_{amp}. As the amplifier gene's maximum transcription rate is chosen to be high, m_{amp} significantly contributes to competition for the cell's translational resources by binding ribosomes in order to be translated into a non-toxic protein p_{amp}. Meanwhile, the sensor gene is constitutive and encodes a repressor protein p_{sens}, which regulates the transcription of the annihilator RNA m_{anti}. Since m_{anti} is an antisense RNA strand binding and inactivating m_{act}, it affects the production of m_{amp}. Therefore, the cell's resource competition landscape is manipulated in response to resource coupling-induced changes in the expression of the constitutively expressed protein p_{sens}. Assuming non-cooperative binding of all transcription factor proteins to the promoter DNA of the genes that are regulated by them, the transcription regulation functions are as follows:

$$F_{sens} \equiv F_{act} \equiv 1, \qquad F_{anti} = \frac{K_{sens}}{K_{sens} + p_{sens}}, \qquad F_{amp} = \frac{p_{act}}{K_{act} + p_{act}} \tag{S120}$$

The ODEs describing the system are given in Equations (S121)-(S127). As for the model's parameters, different genes' copy numbers and maximum transcription rates c_i and α_i can be multiplied together as shown in Supplementary Table S8 to yield quantities that are more informative of the controller's behavior. We thus use the parameters ζ, u, κ, and χ in model equations and in Supplementary Table S9 displaying the values of the model parameters.

$$\dot{m}_{sens} = \zeta\lambda(\epsilon, B) - (\beta_{sens} + \lambda(\epsilon, B))m_{sens} \tag{S121}$$

$$\dot{m}_{anti} = F_{anti}\kappa\lambda(\epsilon, B) - (\beta_{anti} + \lambda(\epsilon, B))m_{anti} - \theta m_{act}m_{anti} \tag{S122}$$

$$\dot{m}_{act} = u\kappa\lambda(\epsilon, B) - (\beta_{act} + \lambda(\epsilon, B))m_{act} - \theta m_{act}m_{anti} \tag{S123}$$

$$\dot{m}_{amp} = F_{amp}\chi\lambda(\epsilon, B) - (\beta_{amp} + \lambda(\epsilon, B))m_{amp} \tag{S124}$$

$$\dot{p}_{sens} = \frac{\epsilon(t^c)}{n_{sens}} \cdot \frac{m_{sens}/k_{sens}}{1 + \frac{1}{1-\bar{\phi}_q}\sum_{j\in\{a,r\}\cup X} m_j/k_j} R - \lambda(\epsilon, B) \cdot p_{sens} \tag{S125}$$

$$\dot{p}_{act} = \frac{\epsilon(t^c)}{n_{act}} \cdot \frac{m_{act}/k_{act}}{1 + \frac{1}{1-\bar{\phi}_q}\sum_{j\in\{a,r\}\cup X} m_j/k_j} R - \lambda(\epsilon, B) \cdot p_{act} \tag{S126}$$

$$\dot{p}_{amp} = \frac{\epsilon(t^c)}{n_{amp}} \cdot \frac{m_{amp}/k_{amp}}{1 + \frac{1}{1-\bar{\phi}_q}\sum_{j\in\{a,r\}\cup X} m_j/k_j} R - \lambda(\epsilon, B) \cdot p_{amp} \tag{S127}$$

We are also interested in observing how the controller mitigates external disturbances in the form of additional competition form other heterologous genes' mRNAs. Here, we investigate the simplest-possible case of one additional heterologous gene *dist*, whose transcription is controlled by a function F_{dist} that rises linearly from 0 (no disturbance) to 1 (full expression of disturbing mRNA) over a brief period of 0.1 h and then stays at $F_{dist} = 1$. This setup models an abrupt increase in the number of mRNAs competing for ribosomes, which the AIF controller is expected to counter by decreasing the concentration of another competing species m_{act}. Since the disturbing gene's transcript and protein are not involved in any additional reactions besides transcription, translation, dilution and mRNA degradation, ODEs describing the dynamics of the corresponding mRNA concentration m_{dist} and protein concentration p_{dist} are trivial and can be readily obtained from (Model VI)'s Equations (S75)-(S76) by replacing the generic x_l index with *dist*:

$$\dot{m_{dist}} = F_{dist}c_{dist}\alpha_{dist}\lambda(\epsilon, B) - (\beta_{dist} + \lambda(\epsilon, B))m_{dist} \tag{S128}$$

$$\dot{p_{dist}} = \frac{\epsilon(t^c)}{n_{dist}} \cdot \frac{m_{dist}/k_{dist}}{1 + \frac{1}{1-\bar{\phi}_q}\sum_{j\in\{a,r\}\cup X} m_j/k_j} R - \lambda(\epsilon, B) \cdot p_{dist} \tag{S129}$$

In the main text's Figure 9, we simulate how our controller enhances the modularity of synthetic gene circuits by conferring an inducible genetic module with robustness to changes in resource availability. This inducible module consists of the transcription activation (TA) factor encoded by the gene *ta*, and the output gene *x*. When bound to an inducer molecule, present in the medium in the concentration f, the transcription activation factor upregulates the output gene's expression cooperatively. Therefore, this module's expression dynamics are given by Equations (S130)-(S133).

$$\dot{m_{ta}} = c_{ta}\alpha_{ta}\lambda(\epsilon, B) - (\beta_{ta} + \lambda(\epsilon, B))m_{ta} \tag{S130}$$

$$\dot{p_{ta}} = \frac{\epsilon(t^c)}{n_{ta}} \cdot \frac{m_{ta}/k_{ta}}{1 + \frac{1}{1-\bar{\phi}_q}\sum_{j\in\{a,r\}\cup X} m_j/k_j} R - \lambda(\epsilon, B) \cdot p_{ta} \tag{S131}$$

$$\dot{m_x} = F_x(f, p_{ta}) \cdot c_x\alpha_x\lambda(\epsilon, B) - (\beta_x + \lambda(\epsilon, B))m_x \tag{S132}$$

$$\dot{p_x} = \frac{\epsilon(t^c)}{n_x} \cdot \frac{m_x/k_x}{1 + \frac{1}{1-\bar{\phi}_q}\sum_{j\in\{a,r\}\cup X} m_j/k_j} R - \lambda(\epsilon, B) \cdot p_x \tag{S133}$$

Here, the output gene's transcription regulation function is given by Equation (S134). The parameters for the disturbing gene and the inducible module are given in Supplementary Table S9 alongside those for the antithetic feedback controller itself.

$$F_x(f, p_{ta}) = \frac{F_{x,0} \cdot K_{dna}^{\eta} + \left(\frac{f}{K_{ind} + f} \cdot p_{ta}\right)^{\eta}}{K_{dna}^{\eta} + \left(\frac{f}{K_{ind} + f} \cdot p_{ta}\right)^{\eta}} \tag{S134}$$

Supplementary Table S8: **Calculation of informative model parameters from gene copy numbers and promoter strengths.**

Variable	Description	Formula	Units
ζ	Total transcription rate of sensor mRNA	$c_{sens}\alpha_{sens}$	nM^*
κ	Maximum total transcription rate of annihilator RNA	$c_{anti}\alpha_{anti}$	nM^*
χ	Maximum total transcription rate of amplifier RNA	$c_{amp}\alpha_{amp}$	nM^*
u	Value of F_{anti} enforced by the controller (has one-to-one correspondence with the set-point p_{sens} value defined by Equation (S120))	$\frac{c_{act}\alpha_{act}}{c_{anti}\alpha_{anti}}$	None

* Transcription rates given in the units of nM because in Equations (S121)-(S124) the mRNA production rate is calculated by multiplying these parameters by the cell growth rate λ (units of h^{-1}).

Supplementary Table S9: **Parameters for simulating the synthetic AIF controller circuit described by Equations** (S123)-(S125)**.** Parameter values were chosen in line with the feasible ranges from Supplementary Table S5.

Parameter	Description	Value	Units
ζ	Total transc. rate of sensor mRNA	$5 \cdot 10^3$	nM
κ	Max. total transc. rate of annihilator RNA	$8 \cdot 10^4$	nM
u	Value of F_{anti} enforced by the controller	0.5	None
χ	Max. total transc. rate of amplifier mRNA	$4 \cdot 10^5$	nM
θ	Actuator-annihilator binding rate	300	$\frac{1}{nM \cdot h}$
K_{sens}	Half-saturation constant for p_{sens}-annihilator promoter DNA binding	$7,000$	nM
K_{amp}	Half-saturation constant for p_{act}-amplifier promoter DNA binding	700	nM
β_i	mRNA degradation rate*	6	h^{-1}
k_i^+	mRNA-ribosome binding rate‡	60	$\frac{1}{nM \cdot h}$
k_i^-	mRNA-ribosome dissociation rate‡	60	h^{-1}
n_i	Number of amino acids in protein p_iℵ	300	aa/nM
	**Disturbance gene**		
c_{dist}	Disturbing gene's copy number	100	nM
α_{dist}	Max. transcription rate for disturbing gene	500	None
	**Inducible genetic module**		
c_{ta}	TA factor gene's copy number	100	nM
α_{ta}	TA factor gene's promoter strength	50	None
c_x	Output gene's copy number	100	nM
α_x	Output gene's promoter strength	50	None
$F_{x,0}$	Baseline function value in absence of inducer	0.1	None
K_{ind}	Half-saturation constant for protein-inducer binding	$1,000$	nM
K_{dna}	Half-saturation constant for the TA factor-promoter DNA binding	$7,000$	nM
η	Cooperativity coefficient for the TA factor-promoter DNA binding	2	None
	**Culture medium**		
σ	Culture medium's nutrient quality	0.5	None

*Same generic value, equal to that for native metabolic genes a, for all genes.

‡Same generic value, equal to that for native metabolic genes a, for all protein-encoding genes *act*, *sens*, *dist*, *ta*, and x. Undefined for the annihilator gene *anti*, as it is not translated.

ℵSame generic value, equal to that for native metabolic genes a. For the annihilator gene *anti*, which is not translated, defined as the number of base triplets in the transcript.

S4.4.2 Estimating the controller's setpoints and operation range

With the actuator's and the annihilator's synthesis rates proportional to u and F_{anti}, the error reacted upon by the antithetic integral feedback is the difference between these two values [36]. According to Equation (S120), the setpoint value of the controlled variable p_{sens}, achieved when the error is equal to zero, is equal to:

$$\overline{p}_{sens} = K_{sens} \cdot \frac{1-u}{u} \tag{S135}$$

However, our model can provide more insights into the controller's performance than just this setpoint concentration of p_{sens}. Namely, knowing the circuit parameter values and using the derivations from Supplementary Note S3, we can calculate the values of the resource competition denominator $\overline{D}$ (quantifying the extent of competition for ribosomes in the cell) and the growth rate $\overline{\lambda}$ enforced by the controller. Furthermore, it is possible to find the controller's operation range boundary – that is, the maximum gene expression burden that it can mitigate.

The dilution and degradation of m_{act} and m_{anti} lead to the phenomenon of "leakiness", which prevents the antithetic integral feedback controller from reaching perfect adaptation. In the idealized case, however, leakiness can be neglected [37], allowing to assume a zero steady-state error. Making this simplification in order to obtain easily calculable analytical estimates for $\overline{D}$ and $\overline{\lambda}$, we obtain:

$$\begin{cases} \dot{m}_{act} \approx \overline{\lambda}\kappa u - \theta \overline{m}_{act}\overline{m}_{anti} = 0 \\ \dot{m}_{anti} \approx \overline{\lambda}\kappa F_{anti} - \theta \overline{m}_{act}\overline{m}_{anti} = 0 \end{cases} \Rightarrow \kappa u = \kappa F_{anti} \Leftrightarrow$$
$$\Leftrightarrow \overline{\phi}_{sens} = \frac{\overline{p}_{sens} n_{sens}}{M} = \frac{n_{sens}}{M} \cdot K_{sens} \cdot \frac{1-u}{u} \tag{S136}$$

According to Equation (S78), it can thus be observed that

$$\frac{n_{sens}}{M} \cdot K_{sens} \cdot \frac{1-u}{u} = \frac{\overline{m}_{sens}/\overline{k}^{NB}_{sens}}{\overline{D}-1} \Leftrightarrow$$
$$\Leftrightarrow \overline{D} - 1 = \frac{\overline{m}_{sens}}{\overline{k}^{NB}_{sens}} \cdot \left(\frac{n_{sens}}{M} \cdot K_{sens} \cdot \frac{1-u}{u} \right)^{-1} \tag{S137}$$

Then, by assuming that the share of idle ribosomes is negligible (likewise to Supple-

mentary Note S3.6), we obtain:

$$\overline{\lambda} = \frac{\overline{\epsilon}^{NB}}{M}\overline{B} \approx \frac{\overline{\epsilon}^{NB}}{M}\overline{R} \Leftrightarrow \overline{\lambda} \approx \frac{\overline{\epsilon}^{NB}}{M} \cdot \frac{M}{n_r} \cdot \frac{\overline{m}_r/\overline{k}_r^{NB}}{\overline{D}-1} \Leftrightarrow$$
$$\Leftrightarrow \overline{\lambda} \approx \frac{\overline{\epsilon}^{NB}}{n_r} \cdot \frac{\overline{m}_r}{\overline{k}_r^{NB}} \cdot \left(\frac{\overline{m}_{sens}}{\overline{k}_{sens}^{NB}}\right)^{-1} \cdot \frac{n_{sens}}{M} \cdot K_{sens} \cdot \frac{1-u}{u} \tag{S138}$$

Now, let us use substitute the formula for steady-state mRNA concentrations from Equation (S97) for the sensor and ribosomal gene transcripts. Assuming the two mRNAs have similar degradation rates, we make the following simplification:

$$\frac{\overline{m}_r}{\overline{m}_{sens}} = \frac{\overline{F}_r^{NB} c_r \alpha_r \overline{\lambda}}{\overline{\lambda} + \beta_r} \cdot \left(\frac{\zeta\overline{\lambda}}{\overline{\lambda} + \beta_{sens}}\right)^{-1} = \frac{\overline{F}_r^{NB} c_r \alpha_r}{\zeta} \tag{S139}$$

Substituting Equation (S139) into Equation (S138), we finally obtain a formula for the steady-state growth rate of the cell:

$$\overline{\lambda} \approx \frac{\overline{\epsilon}^{NB}}{M} \cdot \overline{F}_r^{NB} c_r \alpha_r \cdot \frac{n_{sens}\overline{k}_{sens}^{NB}}{n_r \overline{k}_r^{NB}} \cdot \frac{K_{sens}}{\zeta} \cdot \frac{1-u}{u} \tag{S140}$$

Knowing the growth rate, from Equation (S121) we can find the steady-state value of the sensor gene mRNA as

$$\overline{m}_{sens} = \frac{\overline{\lambda}\zeta}{\overline{\lambda} + \beta_{sens}} \tag{S141}$$

which in turn can be plugged into Equation (S137) to yield an estimate for the value of $\overline{D}$ — that is, the extent of resource competition maintained by the controller:

$$\overline{D} = 1 + \frac{\overline{\lambda}\zeta}{(\overline{\lambda} + \beta_{sens})\overline{k}_{sens}^{NB}} \cdot \left(\frac{n_{sens}}{M} \cdot K_{sens} \cdot \frac{1-u}{u}\right)^{-1} \tag{S142}$$

Moreover, with the growth rate known, we can find the total steady-state level of the actuator and amplifier mRNAs. This is useful because the controller combats the effects of the appearance of extra competing heterologous mRNAs by decreasing m_{amp}. The abundance of the actuator mRNA m_{act} is likewise decreased in this case, albeit this has more relevance for regulating the amplifier gene's expression via p_{act} levels rather than for its direct contribution to resource competition, which is small relatively to that of m_{amp}. The maximum possible total concentrations of m_{amp} and m_{act} are thus achieved in the case of no heterologous gene expression. Meanwhile, since the actuator mRNA level

cannot be decreased below zero, the value of

$$max\left(\frac{m_{act}}{k_{act}}+\frac{m_{amp}}{k_{amp}}\right)$$

defines the controller's operation range. By recalling Equations (S136), (S78) and (S97), we can find this sum for the case of no heterologous genes being expressed (besides those comprising the controller). Yielded by the formula in Equation (S143), it gives rise to the definition of our controller's operating range in the main text.

$$\frac{n_{sens}}{M}\cdot K_{sens}\cdot\frac{1-u}{u}=(1-\overline{\phi}_q)\cdot\frac{\overline{m}_{sens}/\overline{k}^{NB}_{sens}}{\overline{m}_{act}/\overline{k}^{NB}_{act}+\overline{m}_{amp}/\overline{k}^{NB}_{amp}+\sum_{j\in\{a,r,sens\}}\overline{m}_j/\overline{k}^{NB}_j}\Leftrightarrow$$

$$\Leftrightarrow\frac{\overline{m}_{act}}{\overline{k}^{NB}_{act}}+\frac{\overline{m}_{act}}{\overline{k}^{NB}_{act}}=\frac{(1-\overline{\phi}_q)M\zeta\overline{\lambda}}{K_{sens}n_{sens}\overline{k}^{NB}_{sens}(\overline{\lambda}+\beta_{sens})}\cdot\frac{1-u}{u}-\sum_{j\in\{a,r,sens\}}\frac{\overline{F}^{NB}_j c_j\alpha_j\overline{\lambda}}{\overline{k}^{NB}_j(\overline{\lambda}+\beta_j)}\quad\text{(S143)}$$

S4.4.3 Additional simulations

Here, we provide the outcomes of additional simulations of our controller's behavior besides those given in the main text's Results section. We consider how the controller behaves in presence of time-varying (namely, oscillating) disturbance, as well as how it is affected by parameter uncertainty and the stochasticity of gene expression. Finally, we show how changing the circuit's parameters allows to eliminate the effects of leakiness at the cost of stability. Likewise to the numerical experiments described in the main text, all circuit parameters were taken from Supplementary Table S9 unless stated otherwise.

Supplementary Figure S6 demonstrates our controller's response to a time-varying perturbation in the form of a disturbing gene whose expression oscillates over time (e.g., due a sine wave optogenetic signal being applied to the cell). We simulate this case with the same Ordinary Differential Equations (S121)-(S129) that we used to simulate a constant perturbation in the main text's Figure 7, except that now the disturbing gene's transcription regulation function oscillating between 0 and 1 as defined by

$$F_{dist}(t) = \begin{cases} 0, \text{ if } t < 0 \\ 0.5 + 0.5\text{sin}(\frac{2\pi t}{\Pi} - \frac{\pi}{2}), \text{ otherwise} \end{cases} \tag{S144}$$

where t is the time since the introduction of disturbance, Π is the oscillation period of 2.5 h, and the phase shift of $\frac{\pi}{2}$ ensures that $F_{dist}(0\ h) = 0$ for to ensure the gene transcription regulation function's continuity in time. As it can be seen in Supplementary Figure S6c-e, the antithetic integral feedback controller not only reduces the adaptation error, rendering the controlled variable, cell growth rate, and the resource competition denominator closer to their pre-disturbance variables, but also reduces their fluctuations once the disturbing gene starts being expressed. Indeed, the amplitude of the fluctuations of p_{sens} in the closed-loop system is more than 1.8-fold smaller than that in the open-loop case (i.e., in the case when $c_{anti} = c_{act} = c_{amp} = 0$).

Given that the parameter fit for our cell model has been found to be sloppy (see Supplementary Note S2), it was important to verify that the controller's performance is not significantly affected by parameter uncertainty. To this end, we drew 100 samples from the probability distribution yielded by our MCMC fitting algorithm, reparameterized the model using the sampled parameter values, simulated the circuit's reaction to disturbance

according to Equations (S121)-(S129), and plotted the resultant trajectories in Supplementary Figure S7a–c. It can be seen that the closed-loop system robustly outperforms the open-loop configuration for all 100 combinations of values of the cell model's fitted parameters $\alpha_r : \alpha_a$, $K_\nu = K_\epsilon$, and ν_{max} (while the chloramphenicol-ribosome binding rate constant's value was also sampled from the distribution, it bore no effect on our simulations due to no antibiotic being present in the culture medium). Moreover, across the cases considered, the adaptation error stays remarkably consistent, varying by less than 1%.

To evaluate the controller's stochastic performance, a hybrid tau-leaping simulation was performed as outlined in Supplementary Note S3.3, where the time step was set to $\Delta t = 10^{-6}\ h$ and ODE integration was performed using the `ode15s` function of Matlab R2023b. The outcomes of our simulations are presented in Supplementary Figure S7d–f. Characteristically of the AIF controller motif, while the closed-loop system exhibits greater variance between the stochastic trajectories (especially for D and λ), this is not detrimental to the control objective of reducing the adaptation error that arises due to disturbance [36].

Moreover, in the main text's Results section we mentioned that increasing the actuator and the annihilator genes' synthesis and mutual annihilation rates – that is, κ and θ – can arbitrarily reduce the error between the steady-state value of the controlled variable and its setpoint [37]. However, this can also lead to instability, although the variable's average value will keep converging to the setpoint as the rates increase [38]. As an example of this behavior, in Supplementary Figure S8 we simulated the behavior of a controller whose parameters κ and θ are ten times the values given in Supplementary Table S9 and the disturbing gene's concentration is $c_{dist} = 400\ nM$ as opposed to 100 nM in the main text's Figure 7 (all other parameters' values remain the same). Notably, the pre-disturbance difference between our analytical estimates for λ and D and the simulated values was almost two times smaller than for the controller with the original values of κ and θ (simulations not plotted here but can be obtained by running the script `figS8_orig_kappatheta.m` in our GitHub repository at `https://github.com/KSechkar/rc_e_coli` [35]). For λ, it decreased from 11.08% to only 6.00%, while for D it shrank from 1.66% to 0.85%. Moreover, while disturbance causes the system to oscillate, increasing κ and θ reduces the adaptation error if it is defined as the difference between a variable's mean

value before and after disturbance. Indeed, Figure S8h–j shows that the error amounts to 5.88% for λ and 0.58% for D. With the original κ and θ, while there are no persistent oscillations, the adaptation error comprises 8.17% and 1.22% respectively, which is ≈ 1.4 times greater.

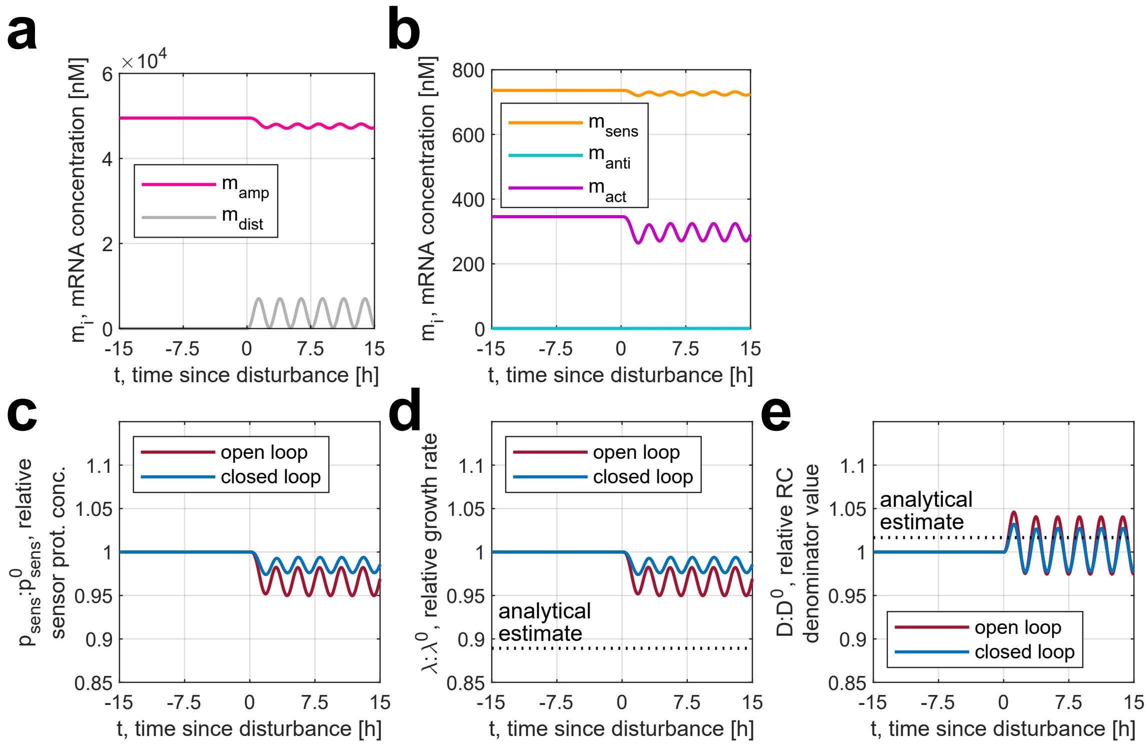

Supplementary Figure S6: **Simulation of the integral controller's response to oscillating disturbance described by Equation** (S144). **a–b** Evolution of mRNA concentrations over time in an example simulation of the first case with the parameters given in Supplementary Table S9. **c–e** The Figures c, d, e show the sensor protein's concentration p_{sens} (the controlled variable), the cell's growth rate λ, and the resource competition denominator D. For comparison, the response of an open-loop system (i.e., in absence of the actuator, the annihilator, and the amplifier genes) is plotted on the same axes. All variables are plotted relative to their steady-state values. Source data are provided as a Source Data file.

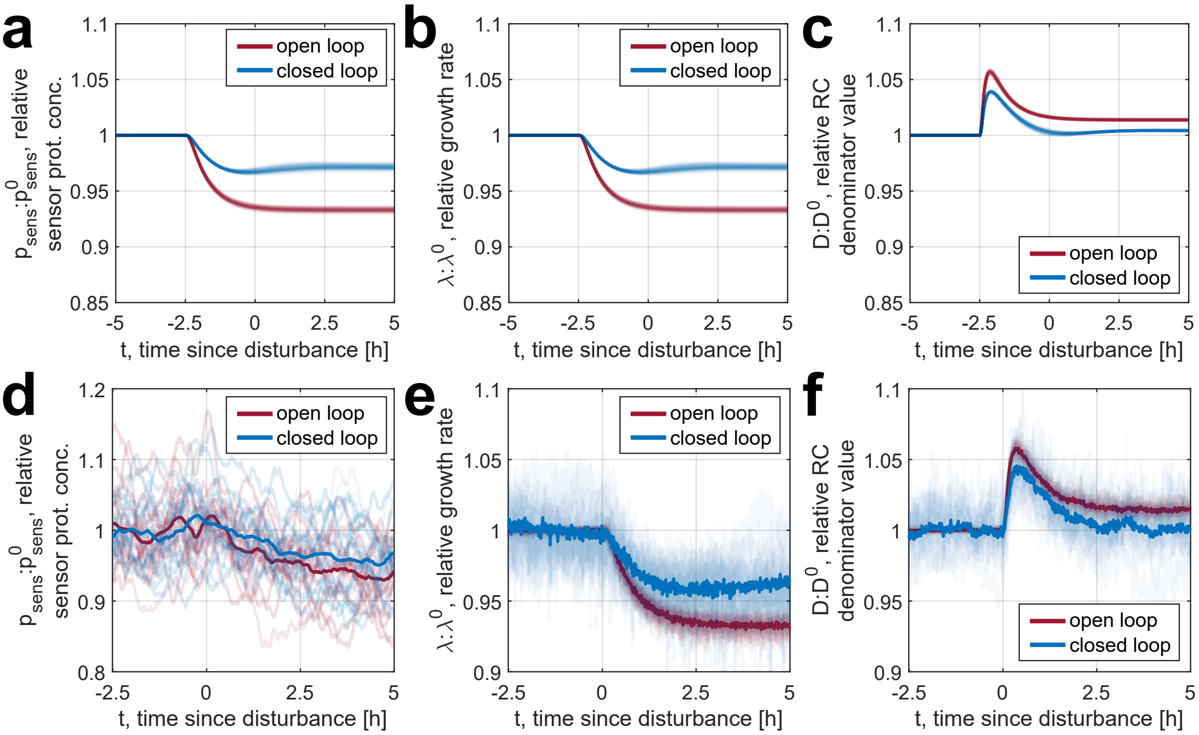

Supplementary Figure S7: **Effect of parameter uncertainty and stochasticity on the integral controller's performance**. **a–c** The sensor protein's concentration p_{sens} (the controlled variable), the cell's growth rate λ, and the resource competition denominator D for 100 different cell model parameter value combinations sampled from the probability distribution obtained by MCMC as outlined in Supplementary Note S2. For comparison, the response of an open-loop system (i.e., in absence of the actuator, the annihilator, and the amplifier genes) is plotted on the same axes. All variables are plotted relative to their steady-state values. **d–f** 48 stochastic trajectories (light blue) of the sensor protein's concentration p_{sens} (the controlled variable), the cell's growth rate λ, and the resource competition denominator D. For comparison, 48 stochastic trajectories of an open-loop system (i.e., in absence of the actuator, the annihilator, and the amplifier genes) is plotted on the same axes. All variables are plotted relative to their steady-state values (light red). The mean trajectories for the closed-and open-loop cases are plotted in bold. Source data are provided as a Source Data file.

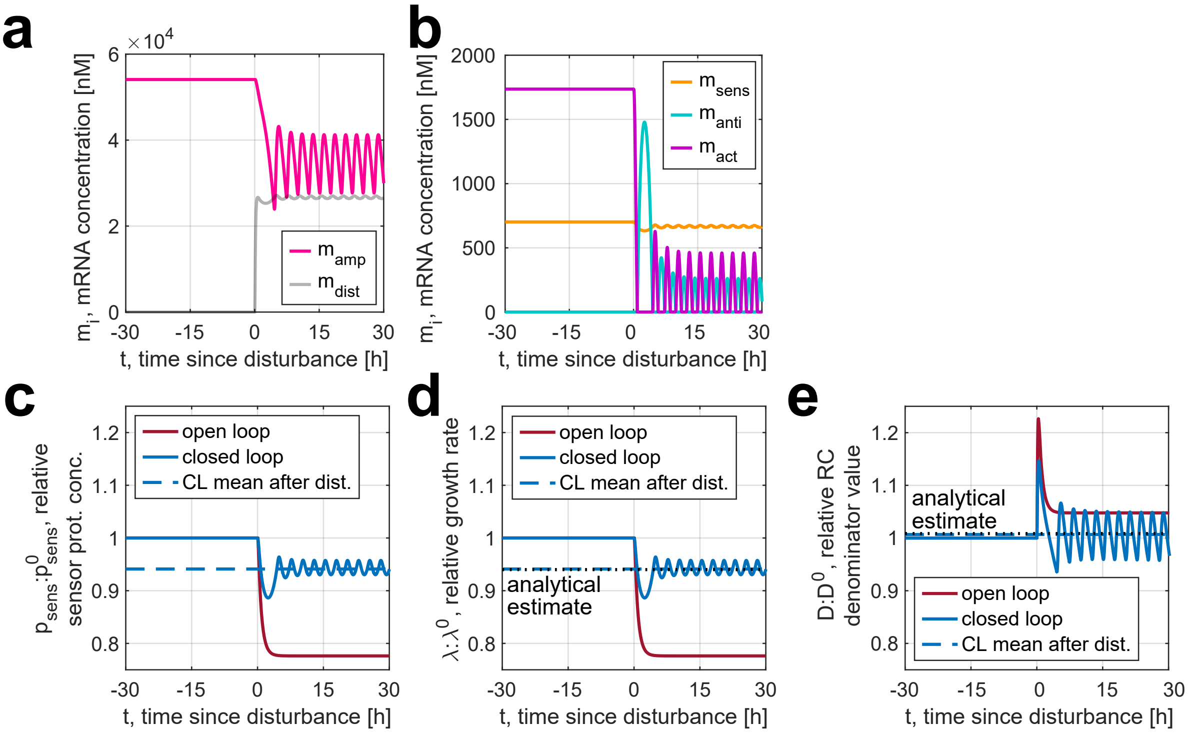

Supplementary Figure S8: **Simulation of the integral controller's response to disturbance with κ and θ increased tenfold. a–b** Evolution of mRNA concentrations over time in an example simulation of the first case with the parameters given in Supplementary Table S9. **c–e** The sensor protein's concentration p_{sens} (the controlled variable), the cell's growth rate λ, and the resource competition denominator D. For comparison, the response of an open-loop system (i.e., in absence of the actuator, the annihilator, and the amplifier genes) is plotted on the same axes. The dashed line represents the average value of a variable over the last 7.5 h of closed-loop simulation. All variables are plotted relative to their steady-state values. Source data are provided as a Source Data file.

References

1. McBride, C. D. & Del Vecchio, D. Predicting Composition of Genetic Circuits with Resource Competition: Demand and Sensitivity. *ACS Synth. Biol.* doi:10.1021/acssynbio.1c00281 (2021).

2. Weiße, A. Y., Oyarzún, D. A., Danos, V. & Swain, P. S. Mechanistic links between cellular trade-offs, gene expression, and growth. *Proc. Natl. Acad. Sci. U.S.A.* **112,** E1038–E1047. doi:10.1073/pnas.1416533112 (2015).

3. Bremer, H. & Dennis, P. P. Modulation of Chemical Composition and Other Parameters of the Cell at Different Exponential Growth Rates. *EcoSal Plus* **3.** doi:10.1128/ecosal.5.2.3 (2008).

4. Hui, S. *et al.* Quantitative proteomic analysis reveals a simple strategy of global resource allocation in bacteria. *Mol. Syst. Biol.* **11,** 784. doi:https://doi.org/10.15252/msb.20145697 (2015).

5. Milo, R. & Phillips, R. *Cell biology by the numbers* (Garland Science, Abingdon, 2015).

6. Chure, G. & Cremer, J. An optimal regulation of fluxes dictates microbial growth in and out of steady-state. *eLife* **12** (ed Bitbol, A.-F.) e84878. doi:10.7554/eLife.84878 (2023).

7. Qian, Y., Huang, H.-H., Jiménez, J. I. & Del Vecchio, D. Resource Competition Shapes the Response of Genetic Circuits. *ACS Synth. Biol.* **6,** 1263–1272. doi:10.1021/acssynbio.6b00361 (2017).

8. Erickson, D. W. *et al.* A global resource allocation strategy governs growth transition kinetics of *Escherichia coli*. *Nature* **551,** 119–123. doi:10.1038/nature24299 (2017).

9. Korem Kohanim, Y. *et al.* A Bacterial Growth Law out of Steady State. *Cell Rep.* **23,** 2891–2900. doi:10.1016/j.celrep.2018.05.007 (2018).

10. Scott, M., Gunderson, C. W., Mateescu, E. M., Zhang, Z. & Hwa, T. Interdependence of Cell Growth and Gene Expression: Origins and Consequences. *Science* **330,** 1099–1102. doi:10.1126/science.1192588 (2010).

11. Vrugt, J. A. Markov chain Monte Carlo simulation using the DREAM software package: Theory, concepts, and MATLAB implementation. *Environ. Model Softw.* **75,** 273–316. doi:10.1016/j.envsoft.2015.08.013 (2016).

12. Klumpp, S., Scott, M., Pedersen, S. & Hwa, T. Molecular crowding limits translation and cell growth. *Proc. Natl. Acad. Sci. U.S.A.* **110,** 16754–16759. doi:10.1073/pnas.1310377110 (2013).

13. Komorowski, M., Costa, M. J., Rand, D. A. & Stumpf, M. P. H. Sensitivity, robustness, and identifiability in stochastic chemical kinetics models. *Proc. Natl. Acad. Sci. U.S.A.* **108,** 8645–8650. doi:10.1073/pnas.1015814108 (2011).

14. Gutenkunst, R. N. *et al.* Universally Sloppy Parameter Sensitivities in Systems Biology Models. *PLoS Comput. Biol.* **3,** e189. doi:10.1371/journal.pcbi.0030189 (2007).

15. Kennell, D. & Riezman, H. Transcription and translation initiation frequencies of the *Escherichia coli* lac operon. *J. Mol. Biol.* **114,** 1–21. doi:https://doi.org/10.1016/0022-2836(77)90279-0 (1977).

16. Scott, M. & Hwa, T. Bacterial growth laws and their applications. *Curr. Opin. Biotechnol.* **22,** 559–565. doi:https://doi.org/10.1016/j.copbio.2011.04.014 (2011).

17. Pine, M. J. Steady-State Measurement of the Turnover of Amino Acid in the Cellular Proteins of Growing *Escherichia coli*: Existence of Two Kinetically Distinct Reactions. *J. Bacteriol.* **103,** 207–215. doi:10.1128/jb.103.1.207-215.1970 (1970).

18. Nath, K. & Koch, A. L. Protein Degradation in *Escherichia coli. J. Biol. Chem.* **245,** 2889–2900. doi:10.1016/s0021-9258(18)63072-8 (1970).

19. Hepp, B., Gupta, A. & Khammash, M. Adaptive hybrid simulations for multiscale stochastic reaction networks. *J. Chem. Phys.* **142,** 034118. doi:10.1063/1.4905196 (2015).

20. Liao, C., Blanchard, A. E. & Lu, T. An integrative circuit–host modelling framework for predicting synthetic gene network behaviours. *Nat. Microbiol.* **2,** 1658–1666. doi:10.1038/s41564-017-0022-5 (2017).

21. El Karoui, M., Hoyos-Flight, M. & Fletcher, L. Future Trends in Synthetic Biology—A Report. *Front. Bioeng. Biotechnol.* **7,** 175. doi:10.3389/fbioe.2019.00175 (2019).

22. Ceroni, F., Algar, R., Stan, G.-B. & Ellis, T. Quantifying cellular capacity identifies gene expression designs with reduced burden. *Nat. Methods* **12,** 415–418. doi:10.1038/nmeth.3339 (2015).

23. Ingram, D. & Stan, G.-B. Modelling genetic stability in engineered cell populations. *Nat. Commun.* **14.** doi:10.1038/s41467-023-39654-4 (2023).

24. Kelly, J. R. *et al.* Measuring the activity of BioBrick promoters using an in vivo reference standard. *Journal of Biological Engineering* **3,** 4. doi:10.1186/1754-1611-3-4 (2009).

25. Hirst, C. D. *Automated BioPart characterisation for synthetic biology* PhD Thesis (Imperial College London, London, 2014).

26. Liu, X. *et al.* De novo design of programmable inducible promoters. *Nucleic Acids Res.* **47,** 10452–10463. doi:10.1093/nar/gkz772 (Sept. 2019).

27. Zucca, S. *et al.* Multi-Faceted Characterization of a Novel LuxR-Repressible Promoter Library for *Escherichia coli. PLOS ONE* **10,** 1–26. doi:10.1371/journal.pone.0126264 (May 2015).

28. Zhang, R. *et al.* Winner-takes-all resource competition redirects cascading cell fate transitions. *Nat. Commun.* **12.** doi:10.1038/s41467-021-21125-3 (2021).

29. Hermsen, R., Erickson, D. W. & Hwa, T. Speed, Sensitivity, and Bistability in Auto-activating Signaling Circuits. *PLoS Comput. Biol.* **7,** 1–9. doi:10.1371/journal.pcbi.1002265 (2011).

30. Tan, C., Marguet, P. & You, L. Emergent bistability by a growth-modulating positive feedback circuit. *Nat. Chem. Biol.* **5,** 842–848. doi:10.1038/nchembio.218 (2009).

31. Balakrishnan, R. *et al.* Principles of gene regulation quantitatively connect DNA to RNA and proteins in bacteria. *Science* **378,** eabk2066. doi:10.1126/science.abk2066 (2022).

32. Paget, M. S. & Helmann, J. D. The σ^{70} family of sigma factors. *Genome Biol.* **4,** 203. doi:10.1186/gb-2003-4-1-203 (2003).

33. Tan, S.-I. & Ng, I.-S. New Insight into Plasmid-Driven T7 RNA Polymerase in *Escherichia coli* and Use as a Genetic Amplifier for a Biosensor. *ACS Synth. Biol.* **9,** 613–622. doi:10.1021/acssynbio.9b00466 (2020).

34. De Cesare, I., Salzano, D., Di Bernardo, M., Renson, L. & Marucci, L. Control-Based Continuation: A New Approach to Prototype Synthetic Gene Networks. *ACS Synth. Biol.* **11,** 2300–2313. doi:10.1021/acssynbio.1c00632 (2022).

35. Sechkar, K., Steel, H., Perrino, G. & Stan, G.-B. *A coarse-grained bacterial cell model for resource-aware analysis and design of synthetic gene circuits* GitHub. 2024. doi:https://doi.org/10.5281/zenodo.10700011.

36. Briat, C., Gupta, A. & Khammash, M. Antithetic Integral Feedback Ensures Robust Perfect Adaptation in Noisy Biomolecular Networks. *Cell Syst.* **2,** 15–26. doi:10.1016/j.cels.2016.01.004 (2016).

37. Qian, Y. & Del Vecchio, D. Realizing 'integral control' in living cells: how to overcome leaky integration due to dilution? *JJ. R. Soc. Interface* **15,** 20170902. doi:10.1098/rsif.2017.0902 (2018).

38. Thorsen, K., Drengstig, T. & Ruoff, P. The effect of integral control in oscillatory and chaotic reaction kinetic networks. *Phys. D: Nonlinear Phenom.* **393,** 38–46. doi:https://doi.org/10.1016/j.physd.2019.01.002 (2019).
