# Supplementary material for: A coarse-grained bacterial cell model for resource-aware analysis and design of synthetic gene circuits
Source: Nat Commun. 2024 Mar 4;15:1981. doi: 10.1038/s41467-024-46410-9 (PMC10912777; doi:10.1038/s41467-024-46410-9)
Supplement: Supplementary file 2 — Reporting Summary [file 41467_2024_46410_MOESM2_ESM.pdf]

nature portfolio

Corresponding author(s): Giansimone Perrino, Guy-Bart Stan

Last updated by author(s): Feb 24, 2024

Reporting Summary

Nature Portfolio wishes to improve the reproducibility of the work that we publish. This form provides structure for consistency and transparency in reporting. For further information on Nature Portfolio policies, see our Editorial Policies and the Editorial Policy Checklist.

Statistics

For all statistical analyses, confirm that the following items are present in the figure legend, table legend, main text, or Methods section.

n/a	Confirmed	
☒	☐	The exact sample size (*n*) for each experimental group/condition, given as a discrete number and unit of measurement
☒	☐	A statement on whether measurements were taken from distinct samples or whether the same sample was measured repeatedly
☒	☐	The statistical test(s) used AND whether they are one- or two-sided<br>*Only common tests should be described solely by name; describe more complex techniques in the Methods section.*
☒	☐	A description of all covariates tested
☒	☐	A description of any assumptions or corrections, such as tests of normality and adjustment for multiple comparisons
☒	☐	A full description of the statistical parameters including central tendency (e.g. means) or other basic estimates (e.g. regression coefficient) AND variation (e.g. standard deviation) or associated estimates of uncertainty (e.g. confidence intervals)
☒	☐	For null hypothesis testing, the test statistic (e.g. *F*, *t*, *r*) with confidence intervals, effect sizes, degrees of freedom and *P* value noted<br>*Give P values as exact values whenever suitable.*
☐	☒	For Bayesian analysis, information on the choice of priors and Markov chain Monte Carlo settings
☒	☐	For hierarchical and complex designs, identification of the appropriate level for tests and full reporting of outcomes
☒	☐	Estimates of effect sizes (e.g. Cohen's *d*, Pearson's *r*), indicating how they were calculated

Our web collection on statistics for biologists contains articles on many of the points above.

Software and code

Policy information about availability of computer code

Data collection	No software was used for data collection in this study.
Data analysis	Matlab R2022a and R2023b (academic use) were used for all numerical simulations and parameter fitting. For Markov Chain Monte Carlo parameter fitting, we employed Matlab Parallel Computing Toolbox v7.6 and the DREAM v2.4 Matlab package, part of the HydroSight v1.3.1 toolbox (https://github.com/peterson-tim-j/HydroSight ; date last accessed: 10 September 2022). Manuscript figures were structured and formatted using Inkscape (v1.2, open source) and Matlab R2022a.<br><br>All scripts and data used to obtain the results described in the manuscript can be found at https://github.com/KSechkar/rc_e_coli

For manuscripts utilizing custom algorithms or software that are central to the research but not yet described in published literature, software must be made available to editors and reviewers. We strongly encourage code deposition in a community repository (e.g. GitHub). See the Nature Portfolio guidelines for submitting code & software for further information.

Data

Policy information about availability of data

All manuscripts must include a data availability statement. This statement should provide the following information, where applicable:

- Accession codes, unique identifiers, or web links for publicly available datasets
- A description of any restrictions on data availability
- For clinical datasets or third party data, please ensure that the statement adheres to our policy

The data generated in this study are provided in the GitHub repository https://github.com/KSechkar/rc_e_coli (ref. [63]) under DOI https://doi.org/10.5281/zenodo.10700011 . The data used for parameter fitting and in Figure 3a and Supplementary Figure 2a are from the study of ref. [19] and are available as Supplementary Material at https://doi.org/10.1126/science.1192588. The data used in Figures 3b–d, Figure 5c, and Supplementary Figure 1 are from the study of ref. [26] and are available in the GitHub repository https://github.com/cremerlab/flux_parity (ref. [89]) under DOI https://doi.org/10.5281/zenodo.5893799 . Source Data are provided with this paper.

Research involving human participants, their data, or biological material

Policy information about studies with human participants or human data. See also policy information about sex, gender (identity/presentation), and sexual orientation and race, ethnicity and racism.

Reporting on sex and gender	No human research participants were involved in this study.
Reporting on race, ethnicity, or other socially relevant groupings	No human research participants were involved in this study.
Population characteristics	No human research participants were involved in this study.
Recruitment	No human research participants were involved in this study.
Ethics oversight	No human research participants were involved in this study.

Note that full information on the approval of the study protocol must also be provided in the manuscript.

Field-specific reporting

Please select the one below that is the best fit for your research. If you are not sure, read the appropriate sections before making your selection.

[x] Life sciences [ ] Behavioural & social sciences [ ] Ecological, evolutionary & environmental sciences

For a reference copy of the document with all sections, see nature.com/documents/nr-reporting-summary-flat.pdf

Life sciences study design

All studies must disclose on these points even when the disclosure is negative.

Sample size	The study did not involve any experimental work or statistical analyses on experimental data, so no sampling from populations of subjects was performed. Sampling design considerations, including the choice of sample sizes, were therefore not applicable.<br><br>When fitting the model's parameters, the dataset from Scott et al. (2010) was used as the one reflecting both empirical "bacterial growth laws" that capture resource allocation in E. coli. Measurements from a single study were taken to exclude the effects of differences between strains and experimental conditions across several distinct experiments.<br><br>When comparing model predictions against experimental results, the dataset from Chure and Cremer (2023) was used. This collection of measurements, spanning over 35 studies into E. coli physiology conducted over the past 55 years, was considered sufficient for a qualitative comparison made between numerical predictions and the extant body of experimental data.
Data exclusions	When fitting the model's parameters, the experimental datapoints, taken from Scott et al. (2010), with growth rates below 0.3/h were excluded from the fitted data set, as the model does not consider the regulatory mechanisms activated in slow-growing cells.<br><br>The cut-off threshold was originally set to be 0.5/h in accordance with Chure and Cremer (2023). This, however, included too few datapoints, so the fitted parameter values did not make the model correctly reproduce the "second bacterial growth law" which postulates an inverse linear relationship between the cell's growth rate and ribosome content as the concentration of chloramphenicol in the culture medium increases (see Scott et al. (2010)). Therefore, the fitting was re-run with the cut-off threshold lowered to 0.3/h, which included at least two different chloramphenicol concentrations for every culture medium involved in the experiment. Post-fitting, this allowed to obtain a second growth law linear regression for every medium type.
Replication	The study did not involve any experimental work. To ensure that the results of this study can be reproduced by independent researchers, all

scripts and data used in this work have been made available online at https://github.com/KSechkar/rc_e_coli

Randomization	The study did not involve any experimental work or statistical analyses on experimental data, so no sampling from populations of subjects was performed. Sampling design considerations, including sample randomisation, were therefore not applicable.
Blinding	No experimental data were collected for this study. Therefore, group allocation was not necessary.

Reporting for specific materials, systems and methods

We require information from authors about some types of materials, experimental systems and methods used in many studies. Here, indicate whether each material, system or method listed is relevant to your study. If you are not sure if a list item applies to your research, read the appropriate section before selecting a response.

Materials & experimental systems

n/a	Involved in the study
☒	☐ Antibodies
☒	☐ Eukaryotic cell lines
☒	☐ Palaeontology and archaeology
☒	☐ Animals and other organisms
☒	☐ Clinical data
☒	☐ Dual use research of concern
☒	☐ Plants

Methods

n/a	Involved in the study
☒	☐ ChIP-seq
☒	☐ Flow cytometry
☒	☐ MRI-based neuroimaging

Plants

Seed stocks	*Report on the source of all seed stocks or other plant material used. If applicable, state the seed stock centre and catalogue number. If plant specimens were collected from the field, describe the collection location, date and sampling procedures.*
Novel plant genotypes	*Describe the methods by which all novel plant genotypes were produced. This includes those generated by transgenic approaches, gene editing, chemical/radiation-based mutagenesis and hybridization. For transgenic lines, describe the transformation method, the number of independent lines analyzed and the generation upon which experiments were performed. For gene-edited lines, describe the editor used, the endogenous sequence targeted for editing, the targeting guide RNA sequence (if applicable) and how the editor was applied.*
Authentication	*Describe any authentication procedures for each seed stock used or novel genotype generated. Describe any experiments used to assess the effect of a mutation and, where applicable, how potential secondary effects (e.g. second site T-DNA insertions, mosiacism, off-target gene editing) were examined.*
